# Supplementary material for: Predicting the environmental suitability and population at risk of podoconiosis in Africa
Source: PLoS Negl Trop Dis. 2020 Aug 27;14(8):e0008616. doi: 10.1371/journal.pntd.0008616 (PMC7480865; doi:10.1371/journal.pntd.0008616)
Supplement: S1 Fig — (PDF) [file pntd.0008616.s002.pdf]

Angola

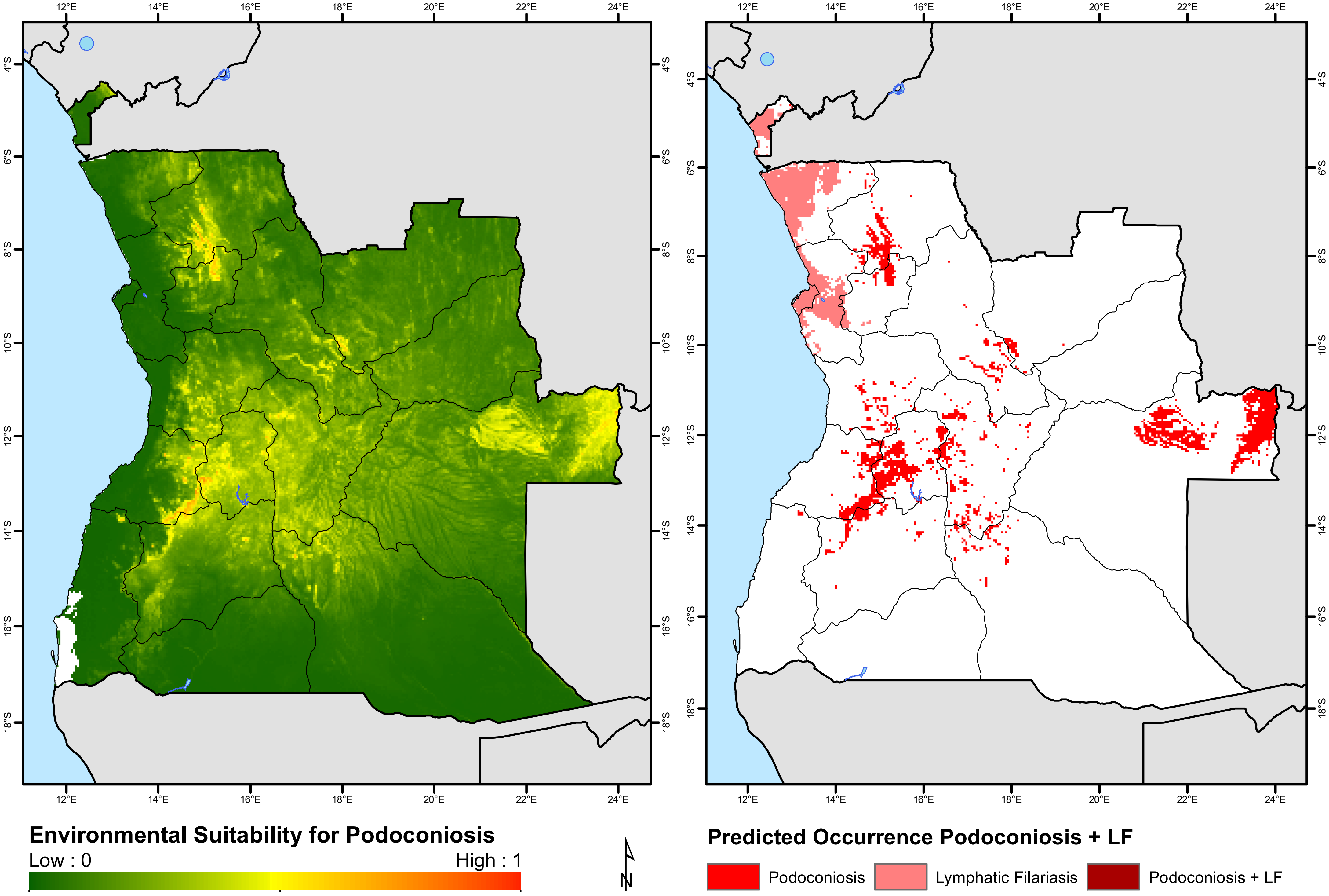
12°E
14°E
16°E
18°E
20°E
22°E
24°E
4°S
6°S
8°S
10°S
12°S
14°S
16°S
18°S
Environmental Suitability for Podoconiosis
Low : 0
High : 1
N
Predicted Occurrence Podoconiosis + LF
Podoconiosis
Lymphatic Filariasis
Podoconiosis + LF

Benin

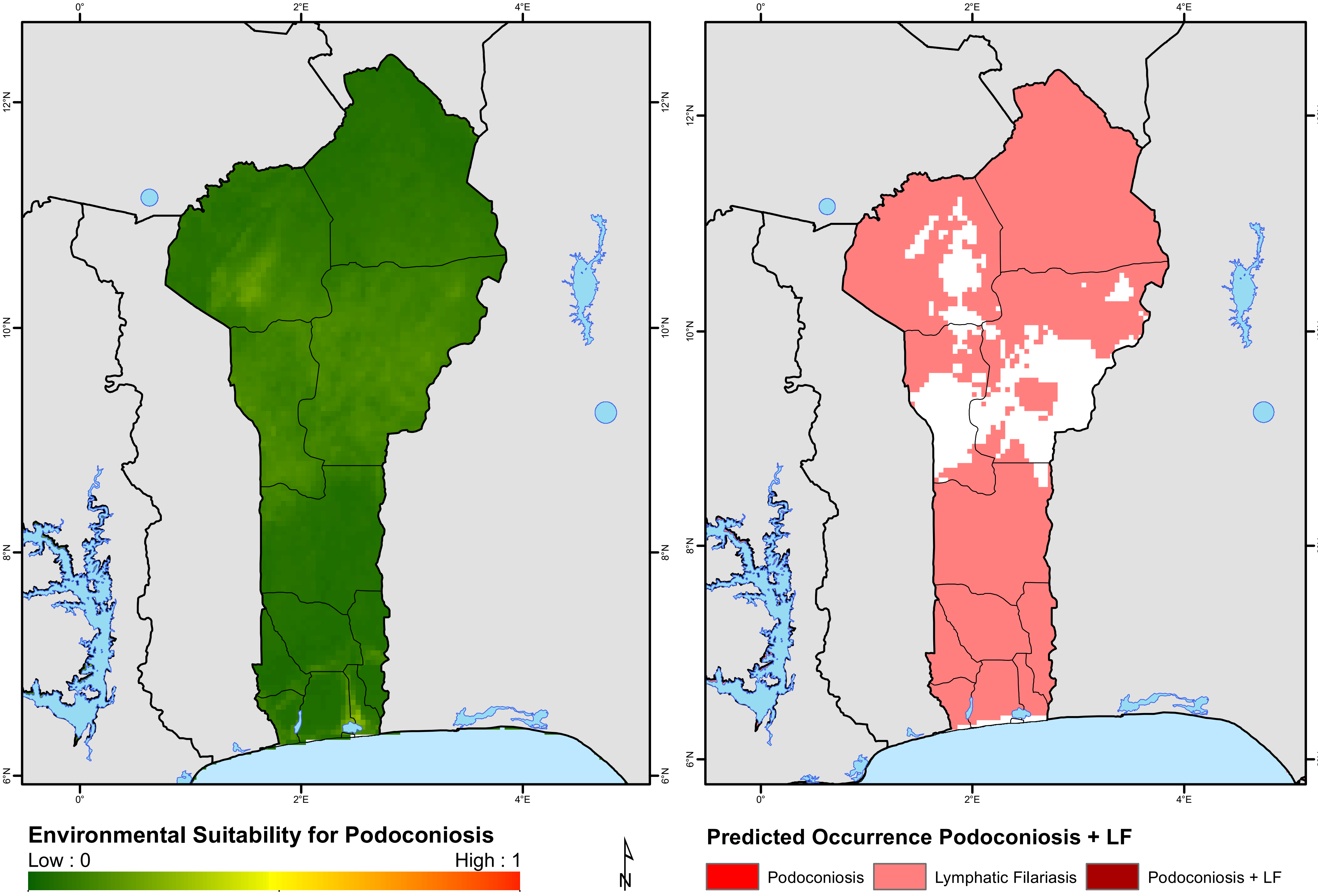
0°
2°E
4°E
12°N
10°N
8°N
6°N
Environmental Suitability for Podoconiosis
Low : 0
High : 1
N
Predicted Occurrence Podoconiosis + LF
Podoconiosis
Lymphatic Filariasis
Podoconiosis + LF

Burkina Faso

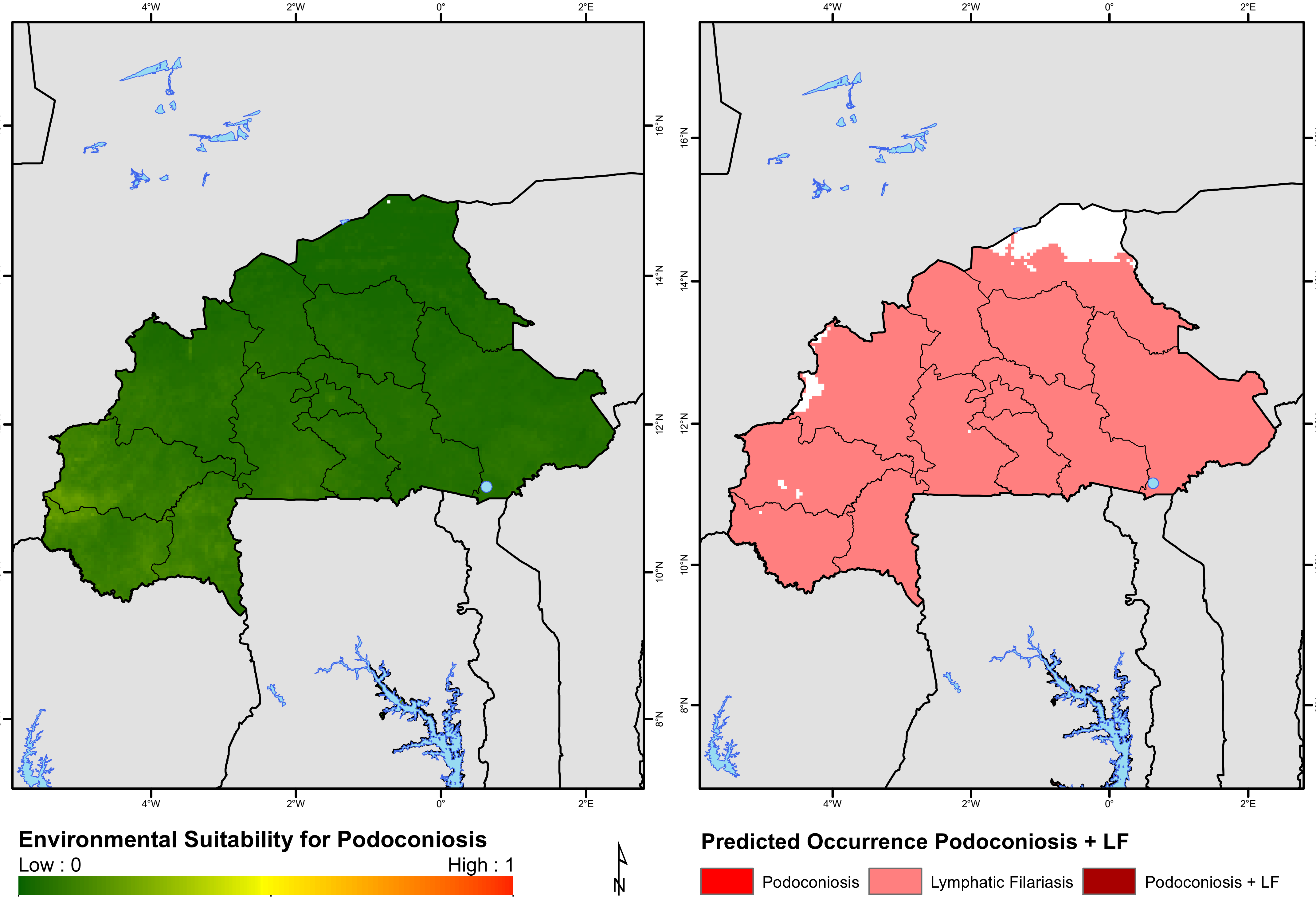

Burundi

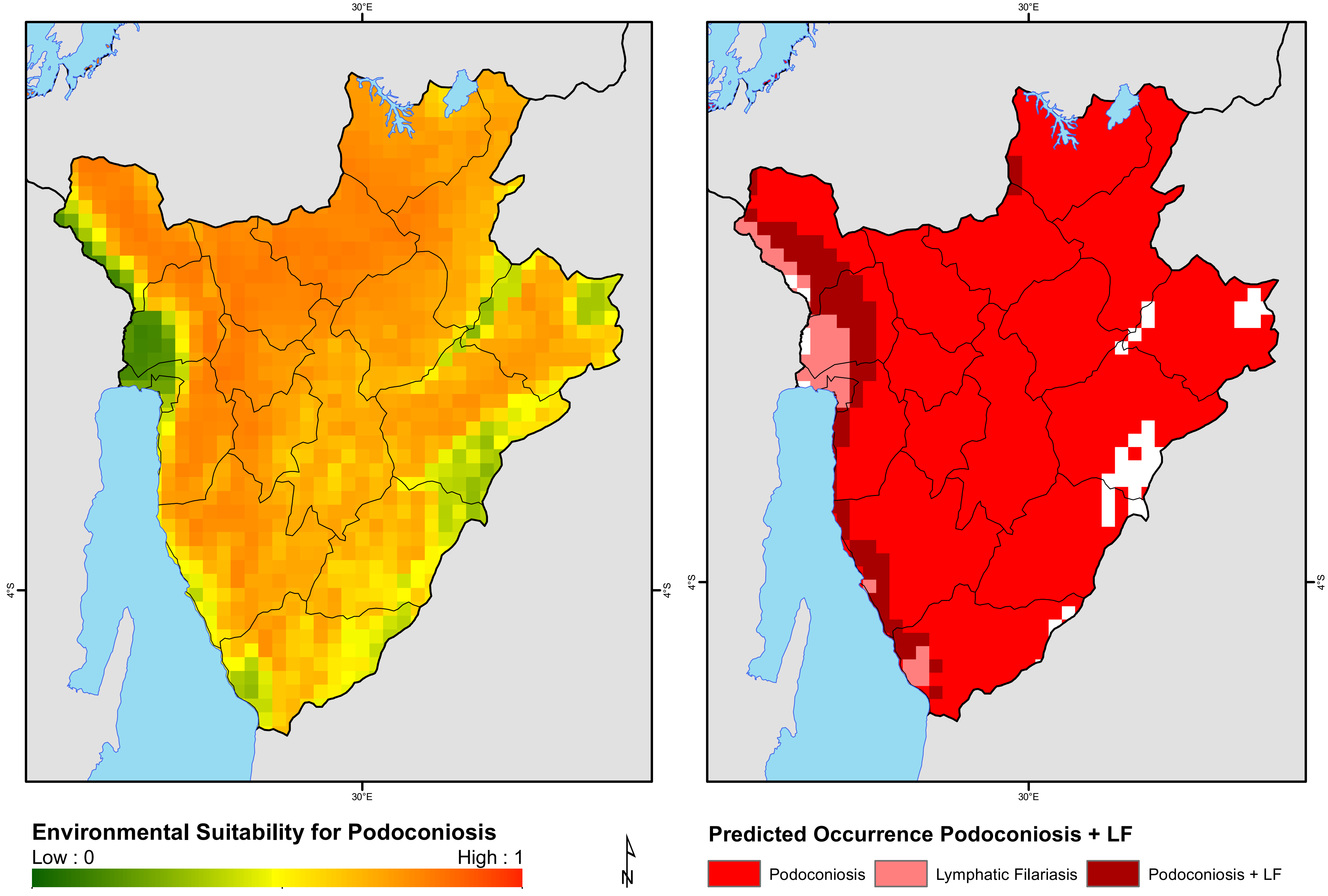
30°E
4°S
Environmental Suitability for Podoconiosis
Low : 0
High : 1
N
Predicted Occurrence Podoconiosis + LF
Podoconiosis
Lymphatic Filariasis
Podoconiosis + LF

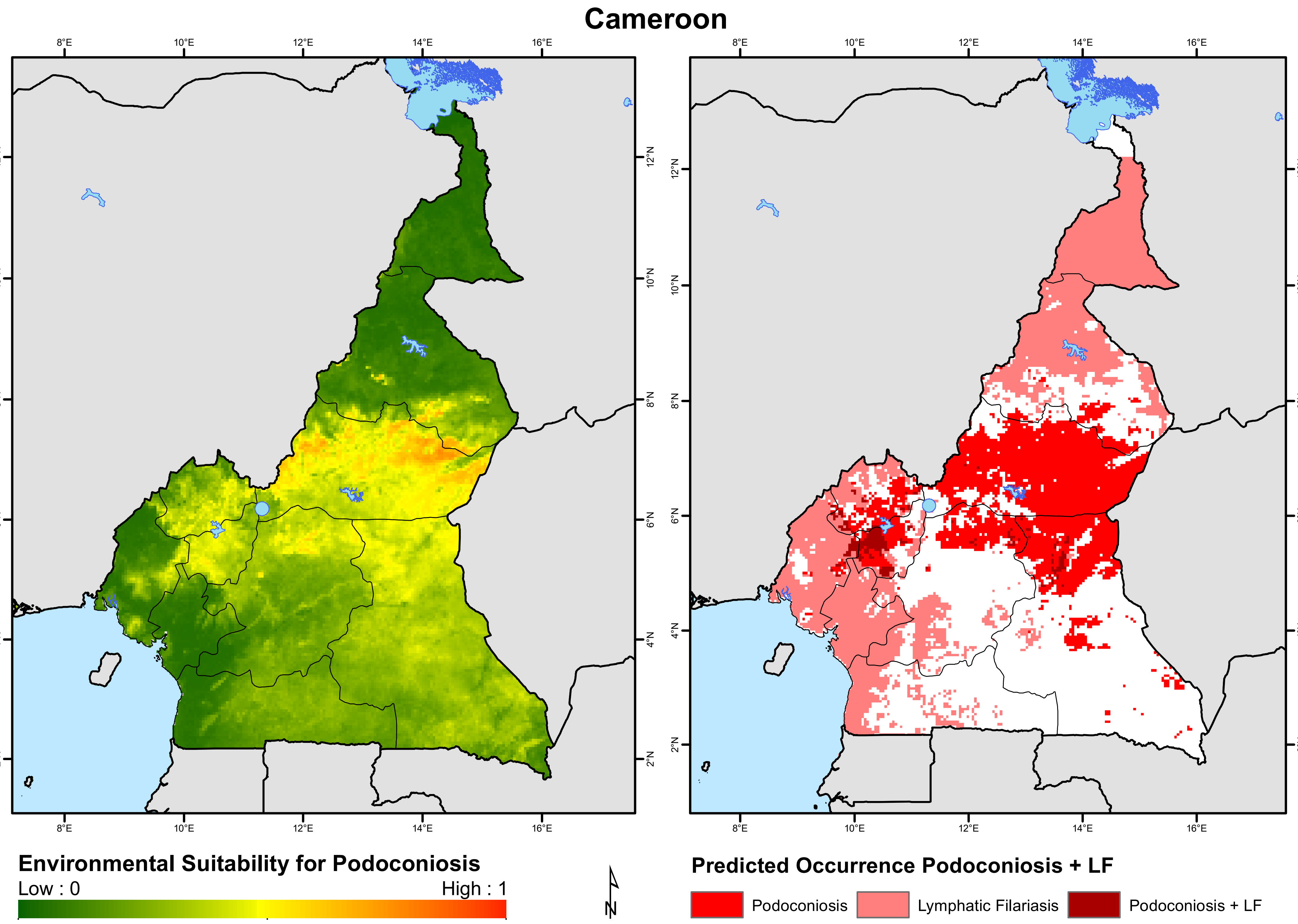
Cameroon
Environmental Suitability for Podoconiosis
Low : 0
High : 1
N
Predicted Occurrence Podoconiosis + LF
Podoconiosis
Lymphatic Filariasis
Podoconiosis + LF

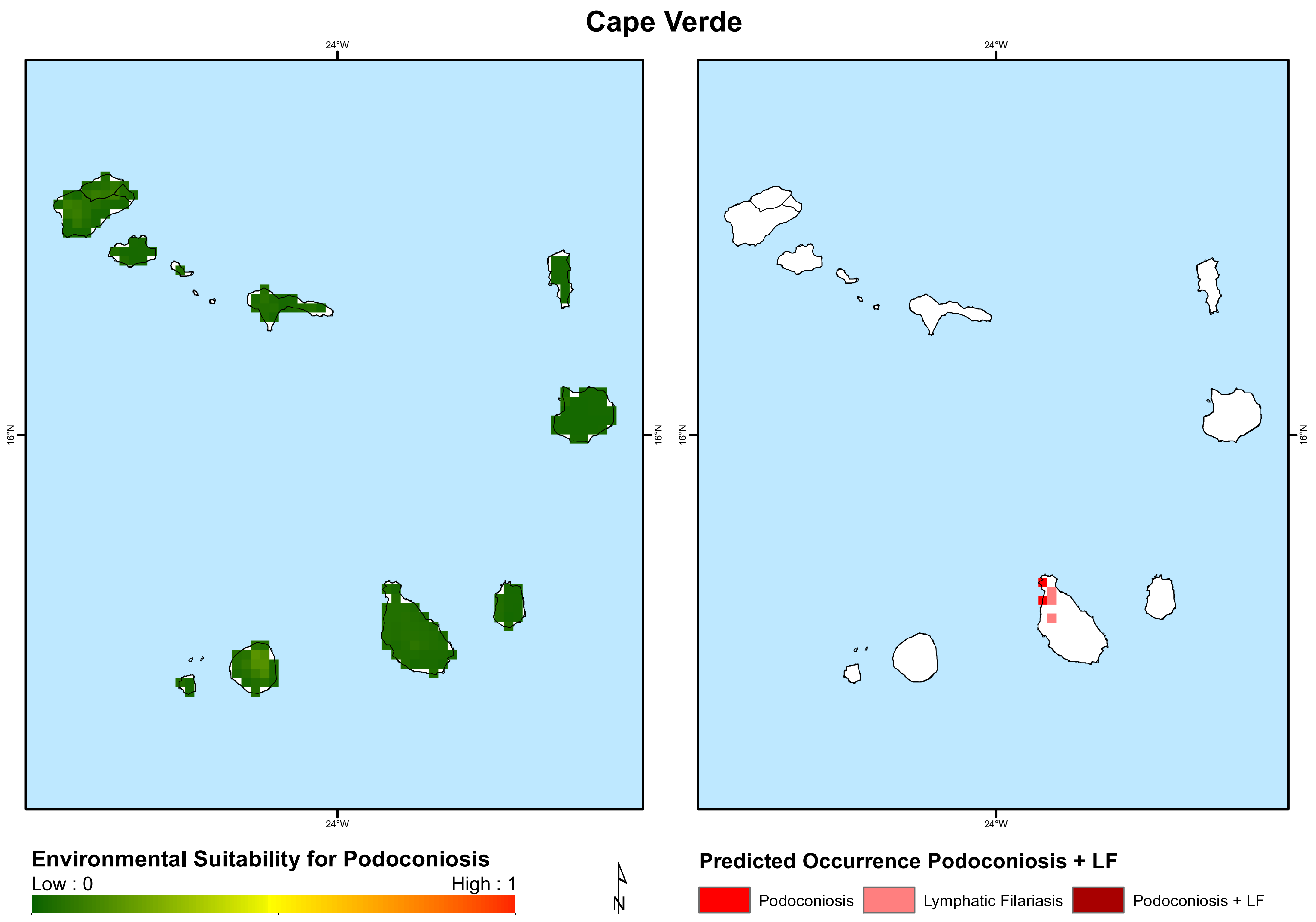
Cape Verde
24°W
16°N
Environmental Suitability for Podoconiosis
Low : 0
High : 1
N
Predicted Occurrence Podoconiosis + LF
Podoconiosis
Lymphatic Filariasis
Podoconiosis + LF

Central African Republic

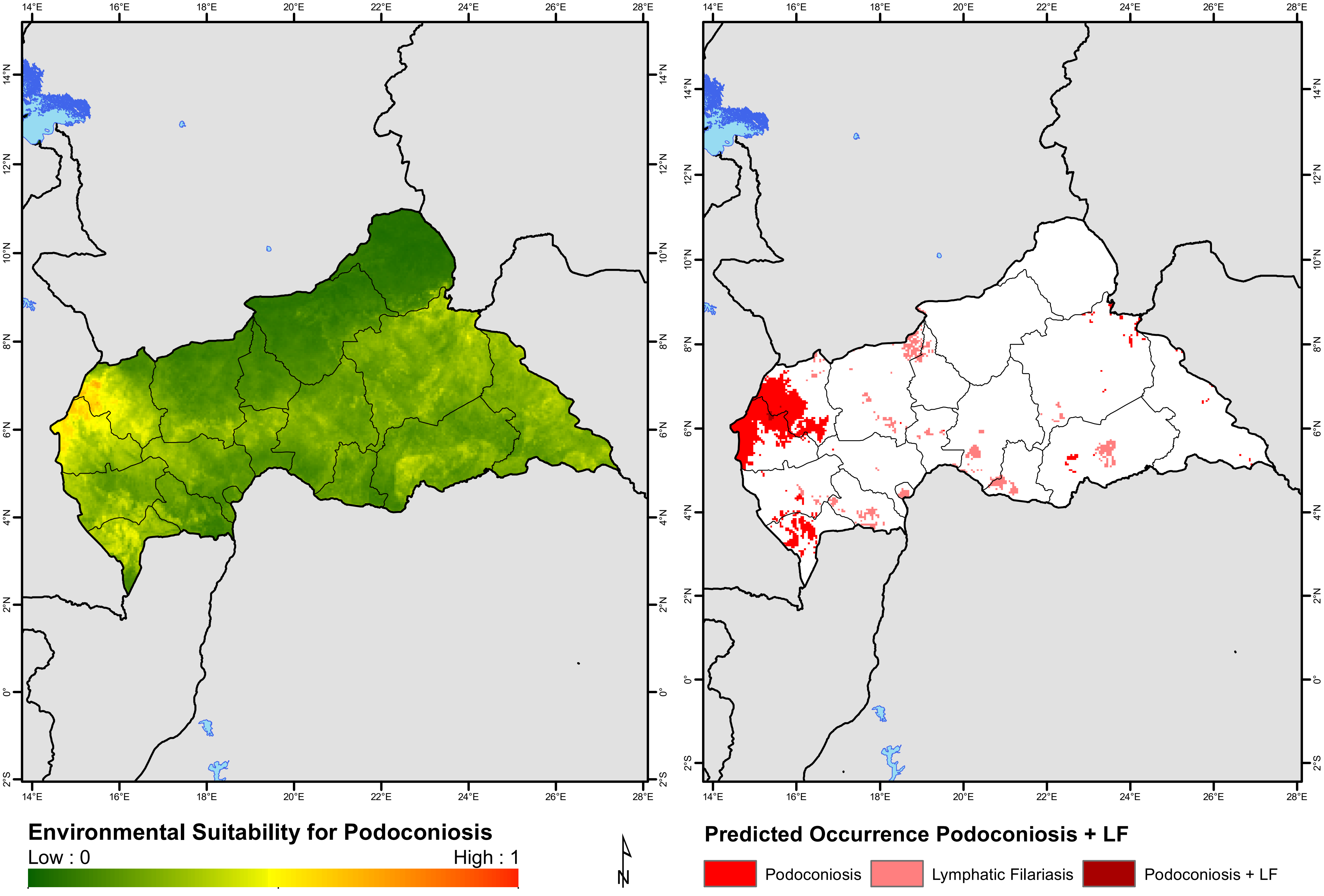

Chad

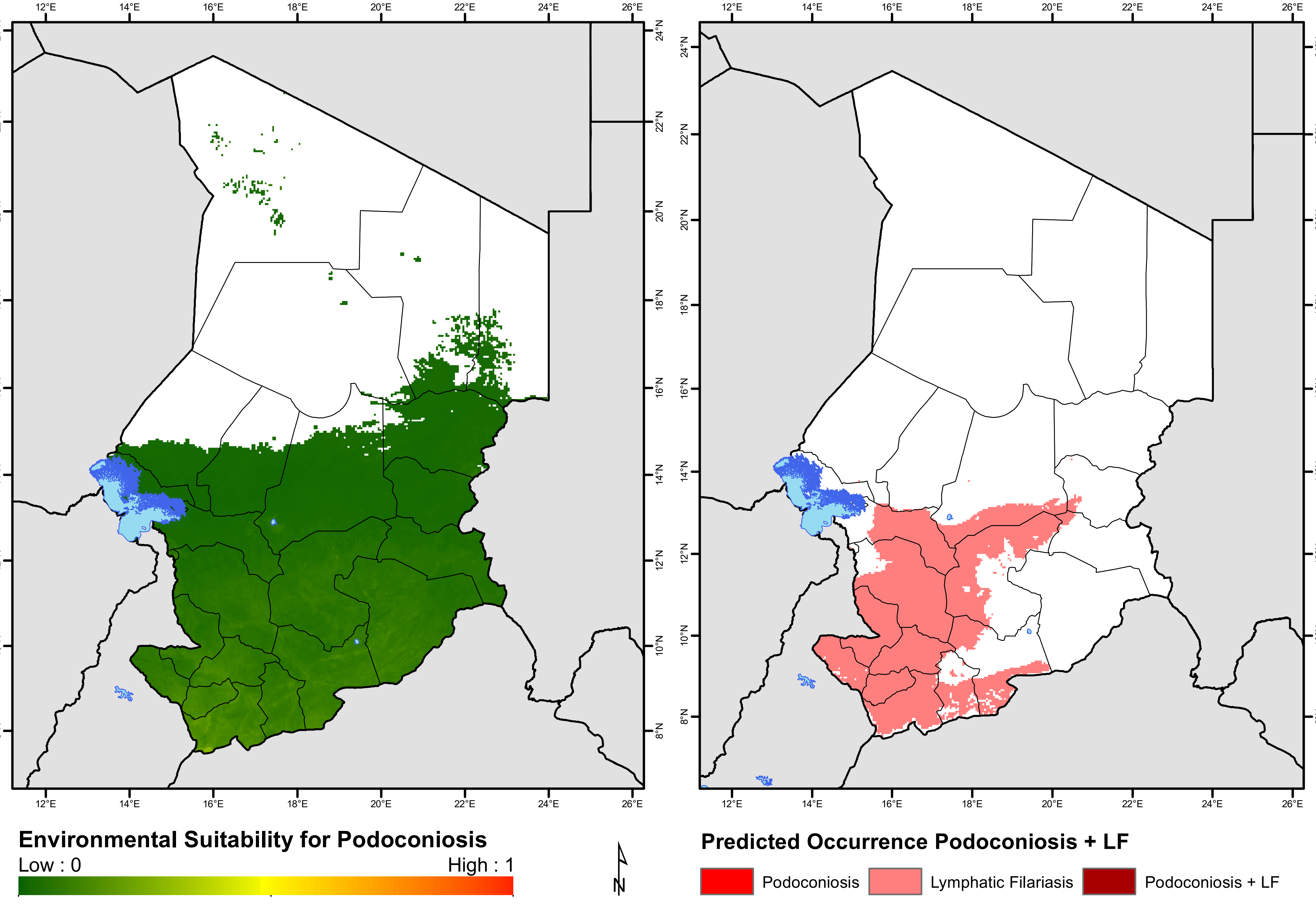
12°E
14°E
16°E
18°E
20°E
22°E
24°E
26°E
8°N
10°N
12°N
14°N
16°N
18°N
20°N
22°N
24°N
Environmental Suitability for Podoconiosis
Low : 0
High : 1
N
Predicted Occurrence Podoconiosis + LF
Podoconiosis
Lymphatic Filariasis
Podoconiosis + LF

Congo

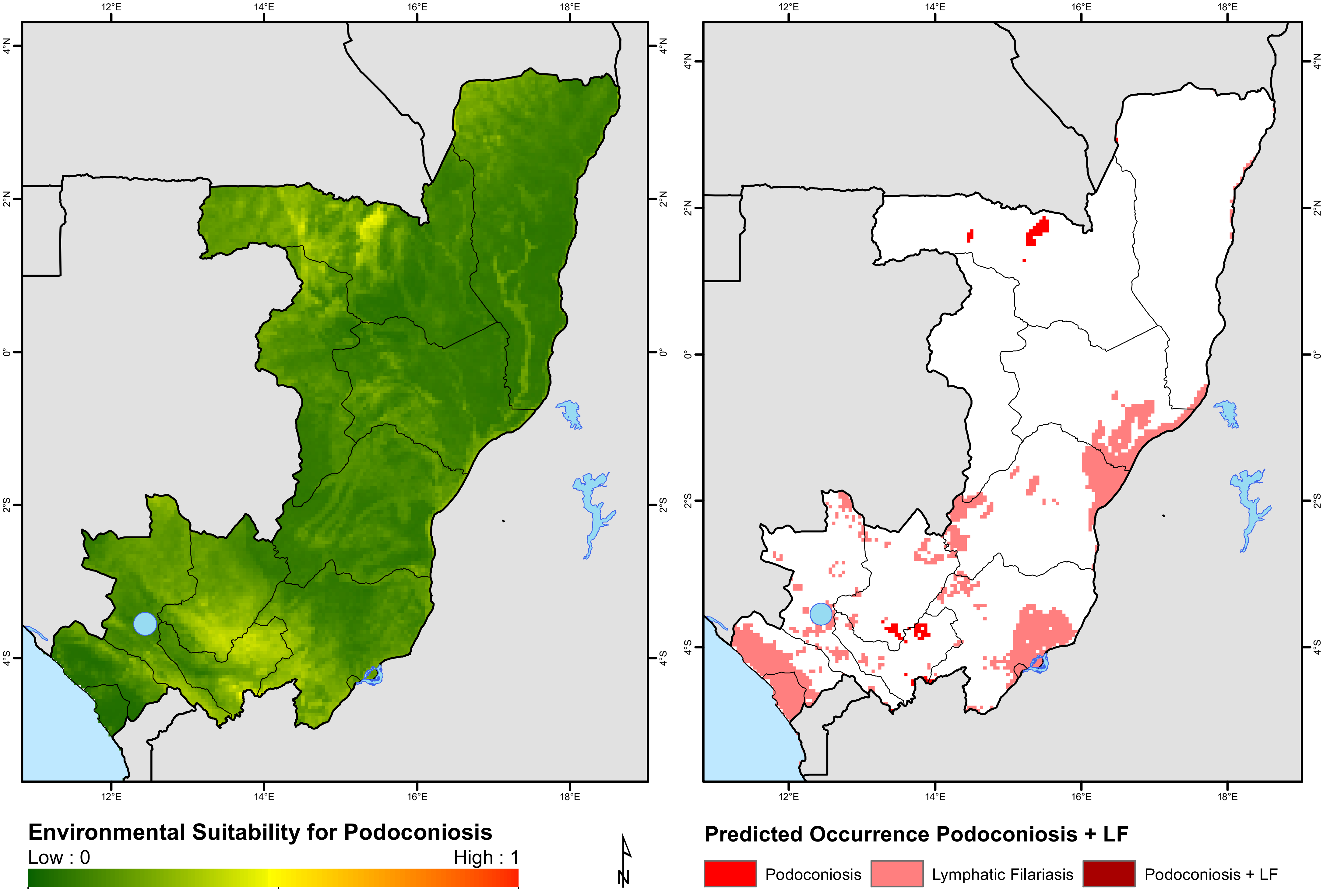
12°E
14°E
16°E
18°E
4°N
2°N
0°
2°S
4°S
Environmental Suitability for Podoconiosis
Low : 0
High : 1
N
Predicted Occurrence Podoconiosis + LF
Podoconiosis
Lymphatic Filariasis
Podoconiosis + LF

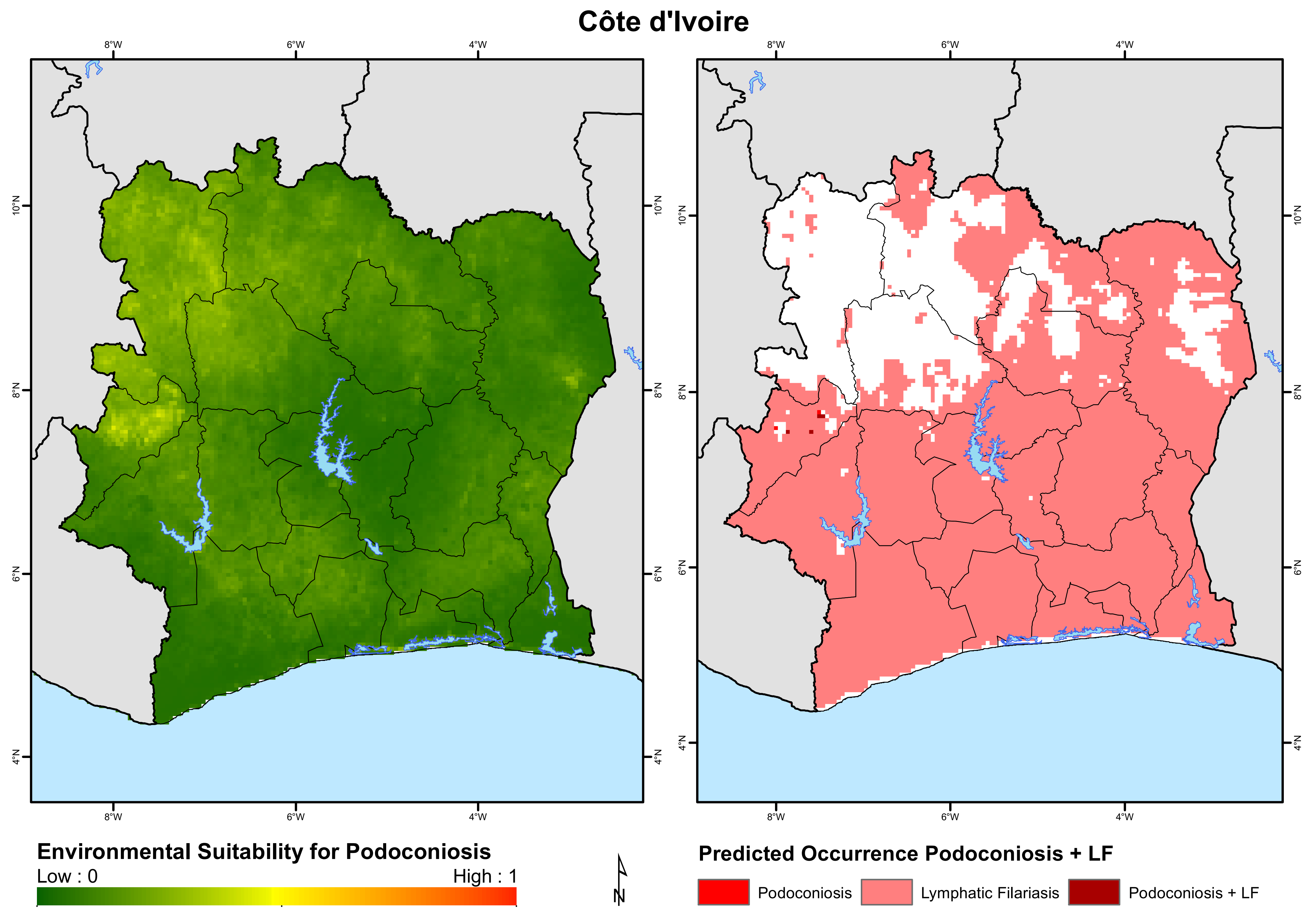

Côte d'Ivoire
8°W
6°W
4°W
10°N
8°N
6°N
4°N
Environmental Suitability for Podoconiosis
Low : 0
High : 1
N
Predicted Occurrence Podoconiosis + LF
Podoconiosis
Lymphatic Filariasis
Podoconiosis + LF

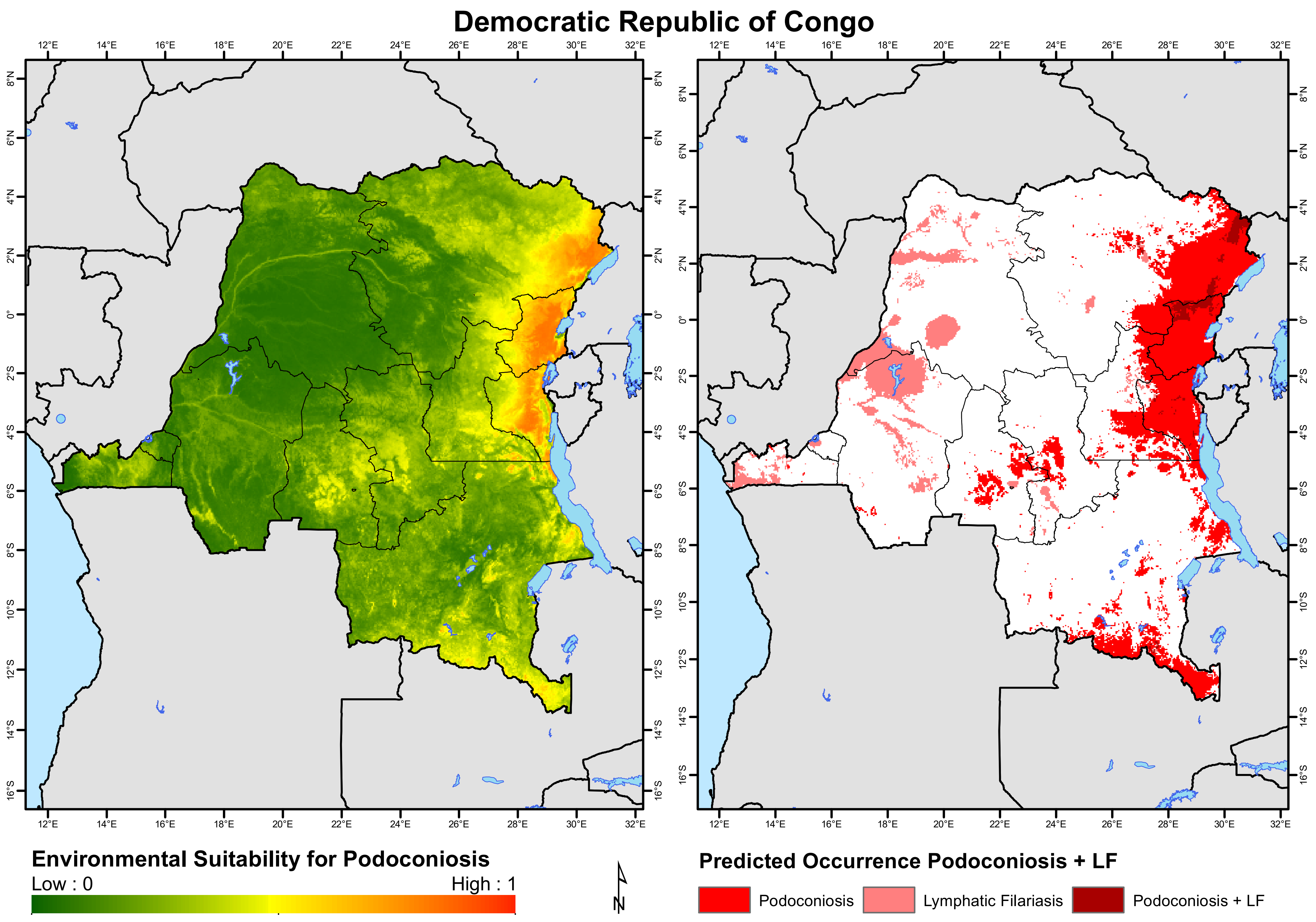
Democratic Republic of Congo
Environmental Suitability for Podoconiosis
Low : 0
High : 1
N
Predicted Occurrence Podoconiosis + LF
Podoconiosis
Lymphatic Filariasis
Podoconiosis + LF

Djibouti

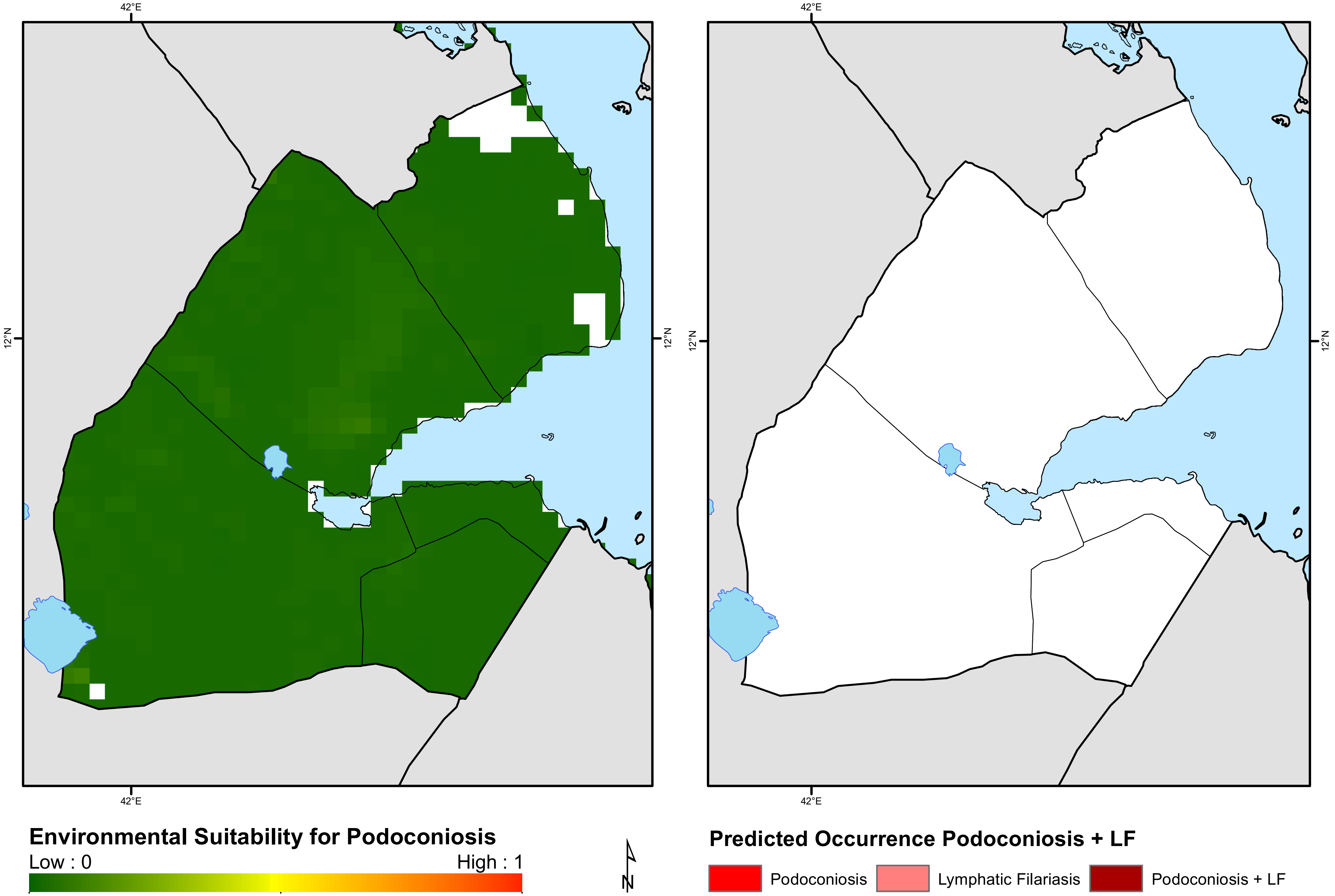
42°E
12°N
Environmental Suitability for Podoconiosis
Low : 0
High : 1
N
Predicted Occurrence Podoconiosis + LF
Podoconiosis
Lymphatic Filariasis
Podoconiosis + LF

Equatorial Guinea

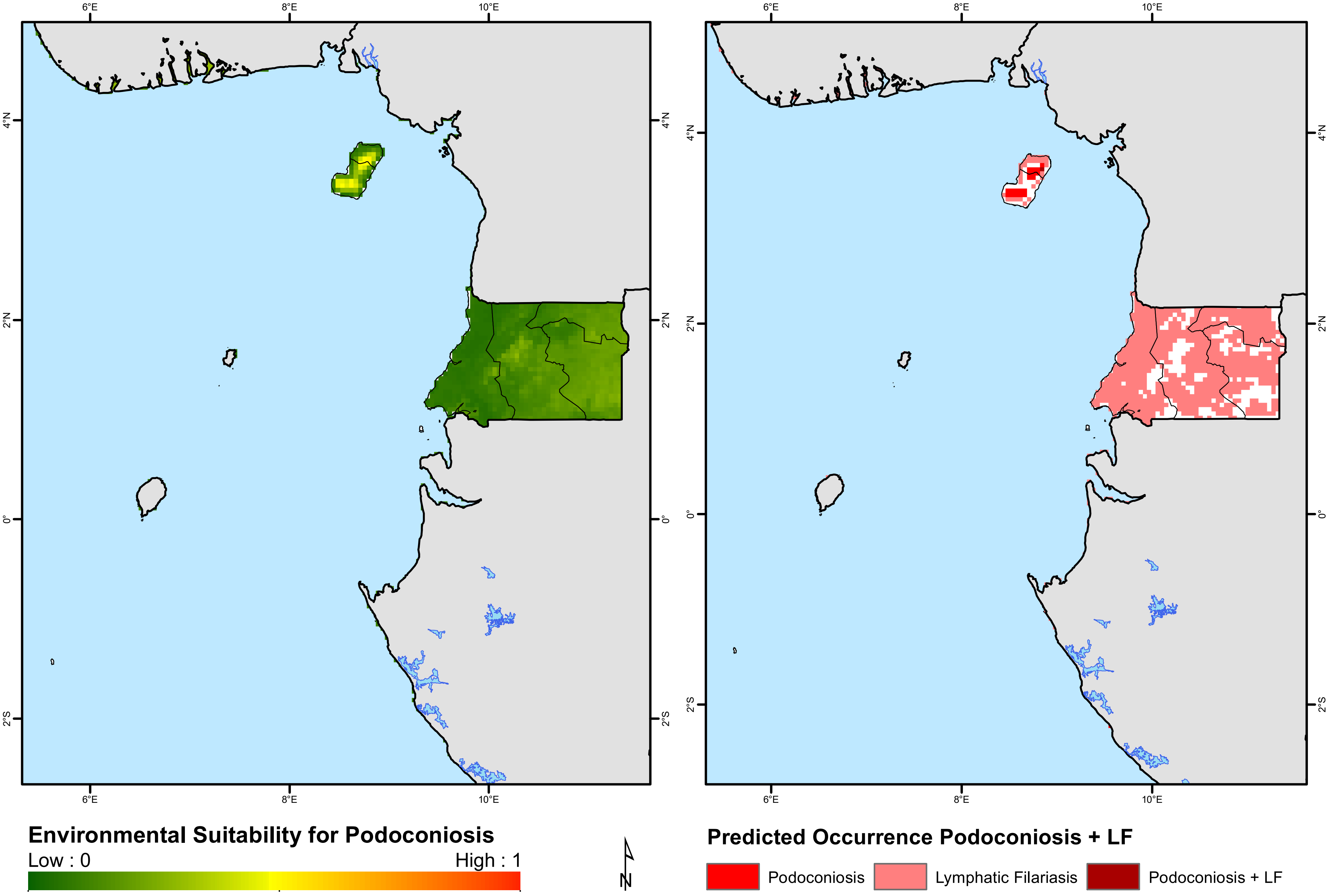

Environmental Suitability for Podoconiosis

Low : 0 High : 1

Predicted Occurrence Podoconiosis + LF

Podoconiosis | Lymphatic Filariasis | Podoconiosis + LF

Eritrea

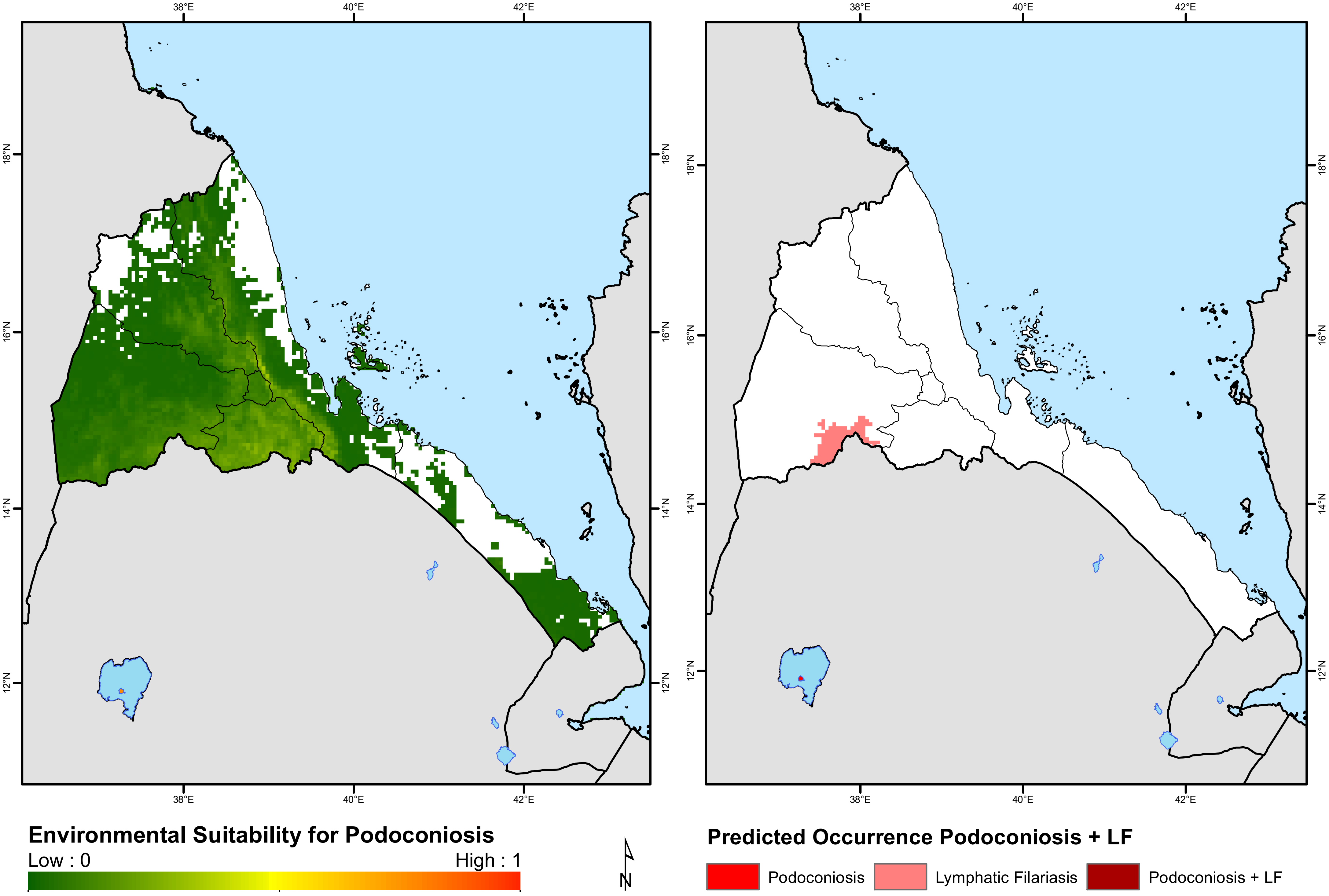

38°E
40°E
42°E
18°N
16°N
14°N
12°N
Environmental Suitability for Podoconiosis
Low : 0
High : 1
N
Predicted Occurrence Podoconiosis + LF
Podoconiosis
Lymphatic Filariasis
Podoconiosis + LF

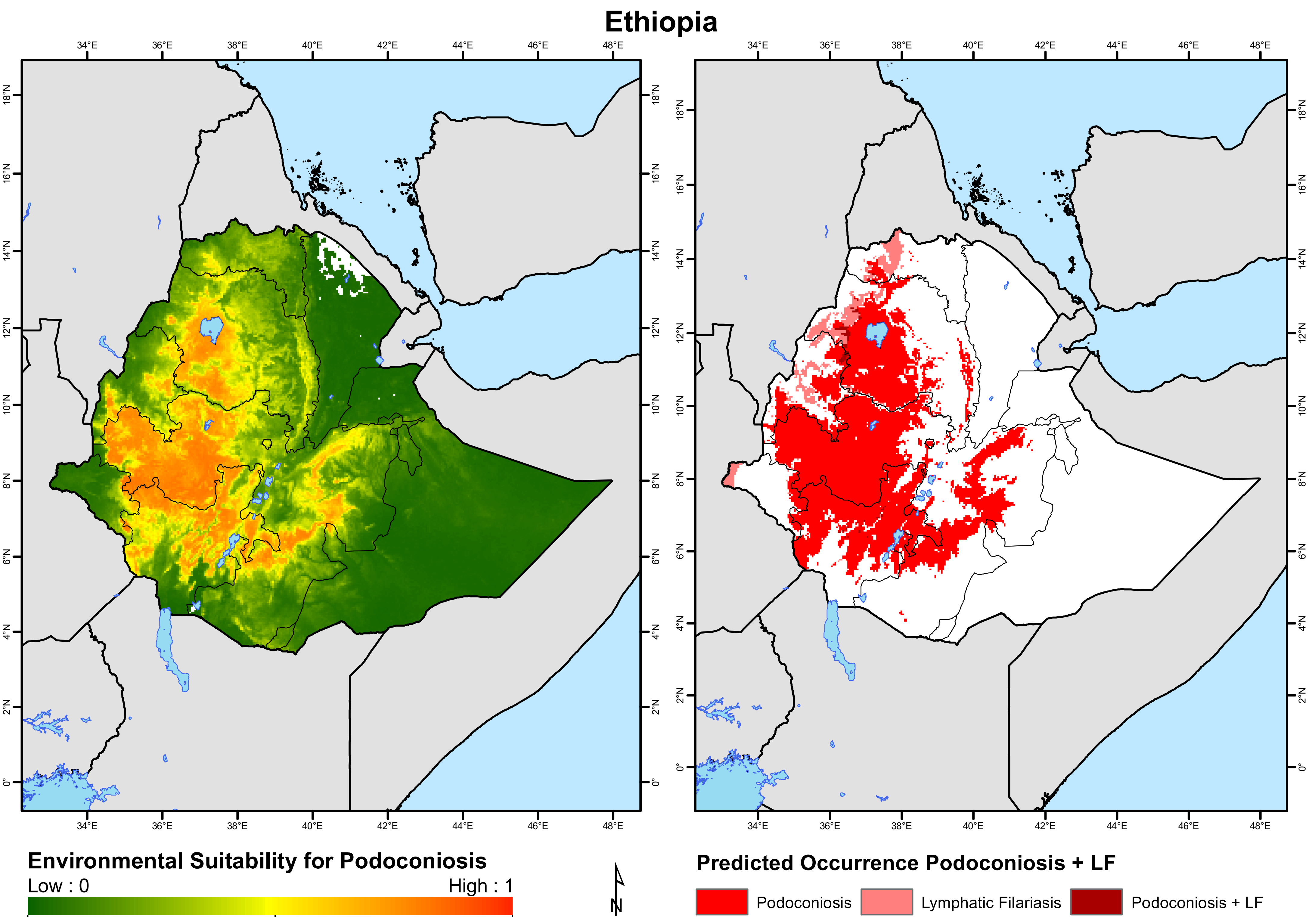
Ethiopia
34°E
36°E
38°E
40°E
42°E
44°E
46°E
48°E
18°N
16°N
14°N
12°N
10°N
8°N
6°N
4°N
2°N
0°
Environmental Suitability for Podoconiosis
Low : 0
High : 1
N
Predicted Occurrence Podoconiosis + LF
Podoconiosis
Lymphatic Filariasis
Podoconiosis + LF

Gabon

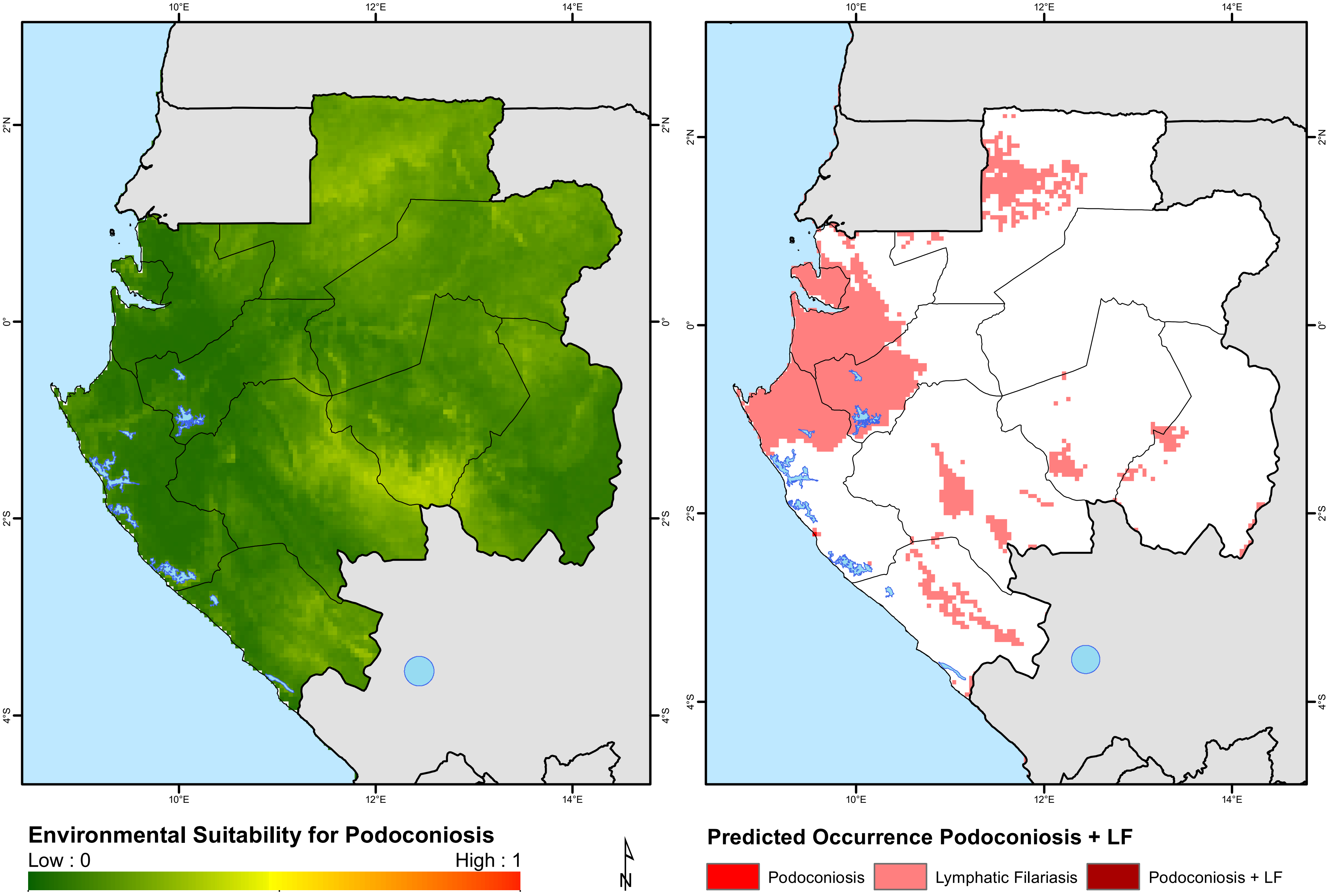
10°E
12°E
14°E
2°N
0°
2°S
4°S
Environmental Suitability for Podoconiosis
Low : 0
High : 1
N
Predicted Occurrence Podoconiosis + LF
Podoconiosis
Lymphatic Filariasis
Podoconiosis + LF

Gambia

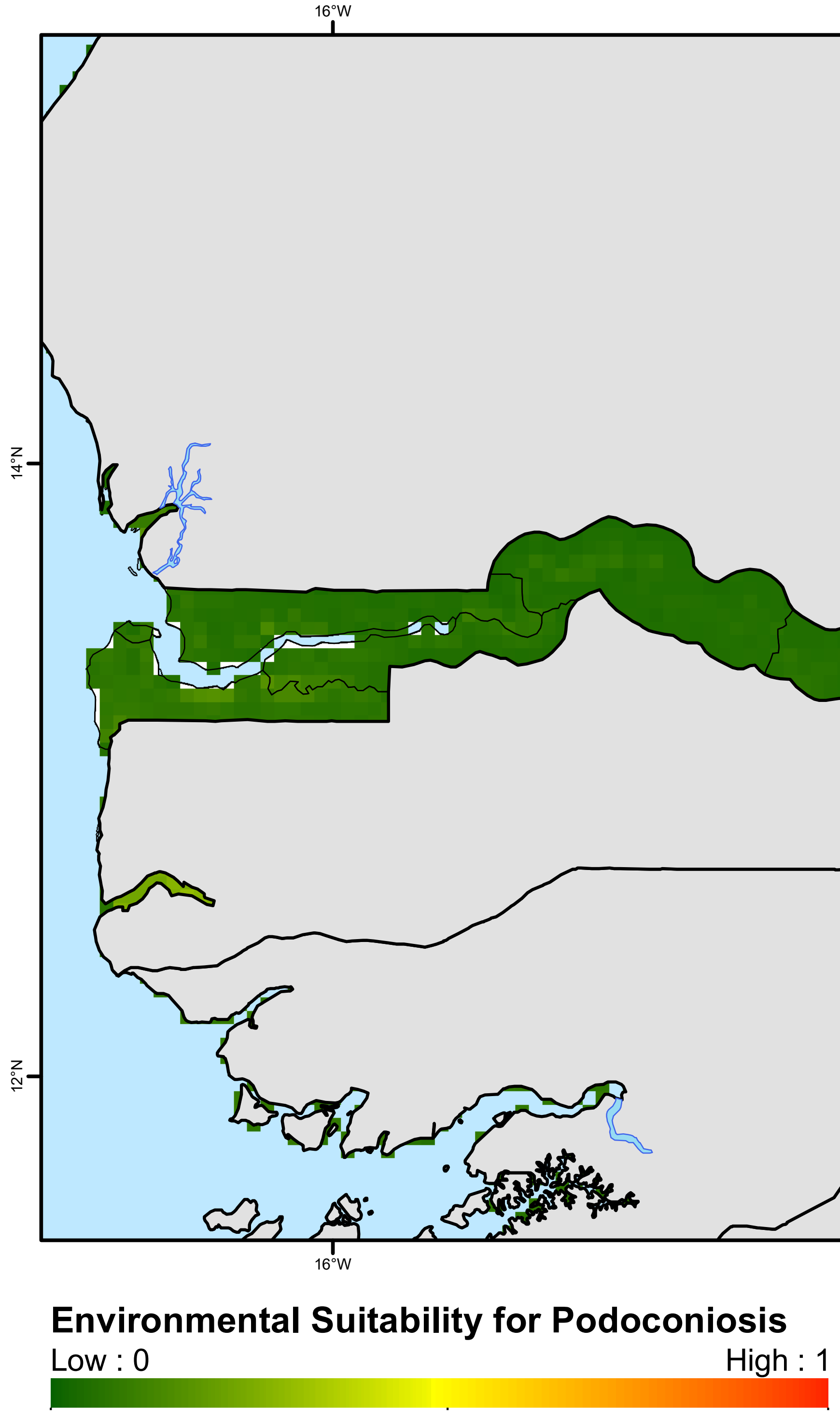

16°W
14°W
14°N
12°N
Environmental Suitability for Podoconiosis
Low : 0
High : 1

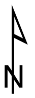

N

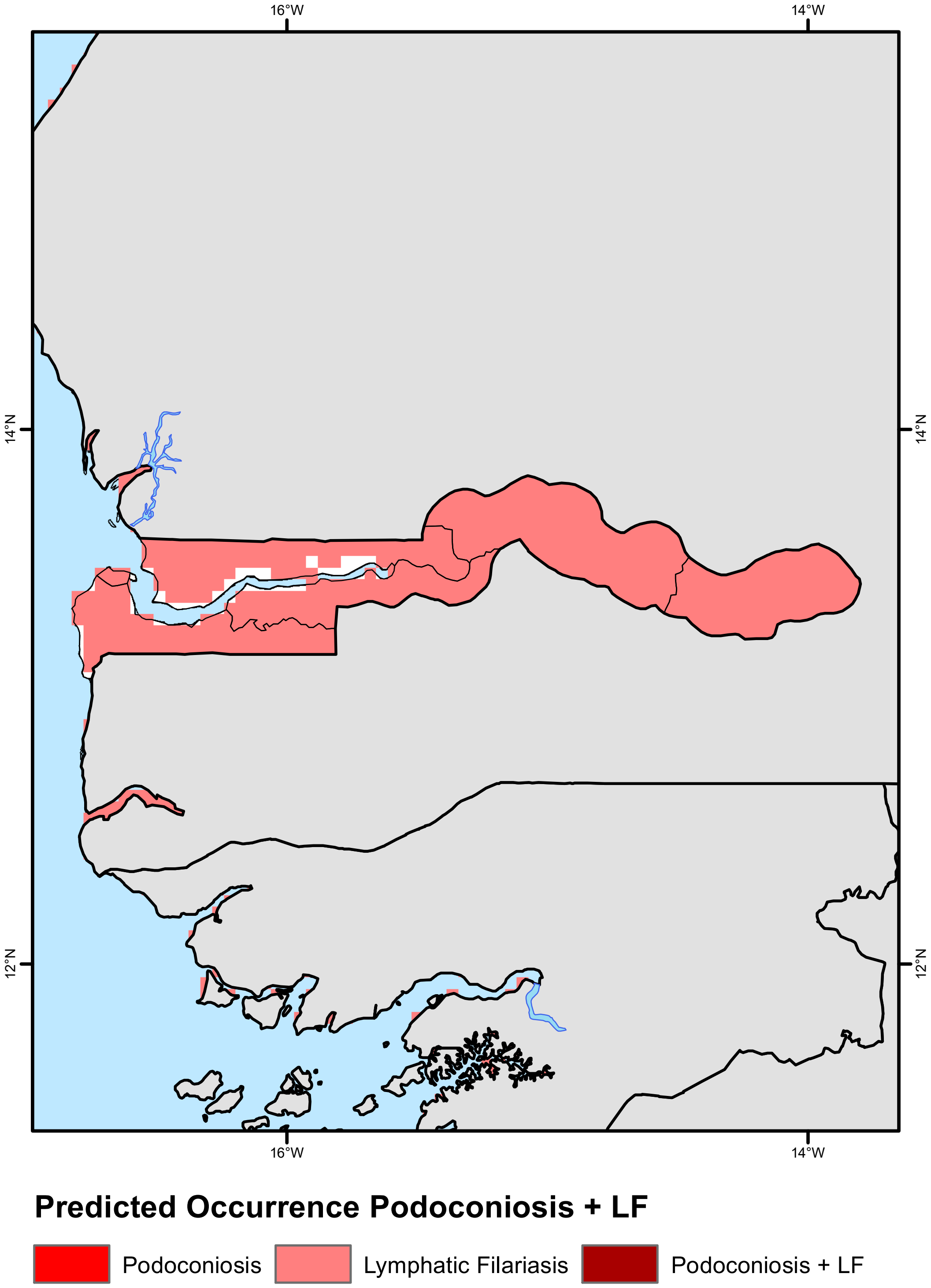

16°W
14°W
14°N
12°N
Predicted Occurrence Podoconiosis + LF
Podoconiosis
Lymphatic Filariasis
Podoconiosis + LF

Ghana

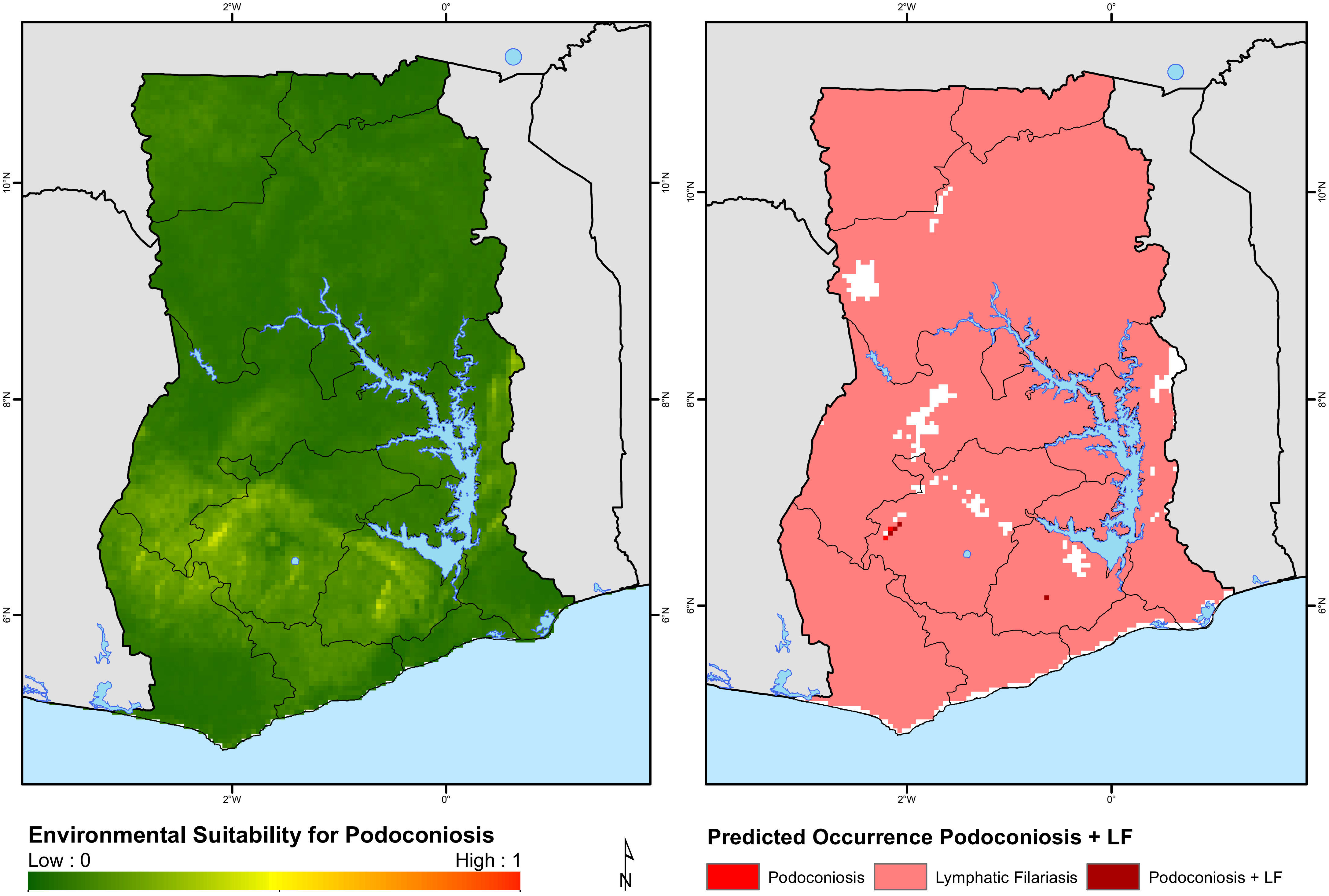

2°W
0°
10°N
8°N
6°N
Environmental Suitability for Podoconiosis
Low : 0
High : 1
N
Predicted Occurrence Podoconiosis + LF
Podoconiosis
Lymphatic Filariasis
Podoconiosis + LF

Guinea

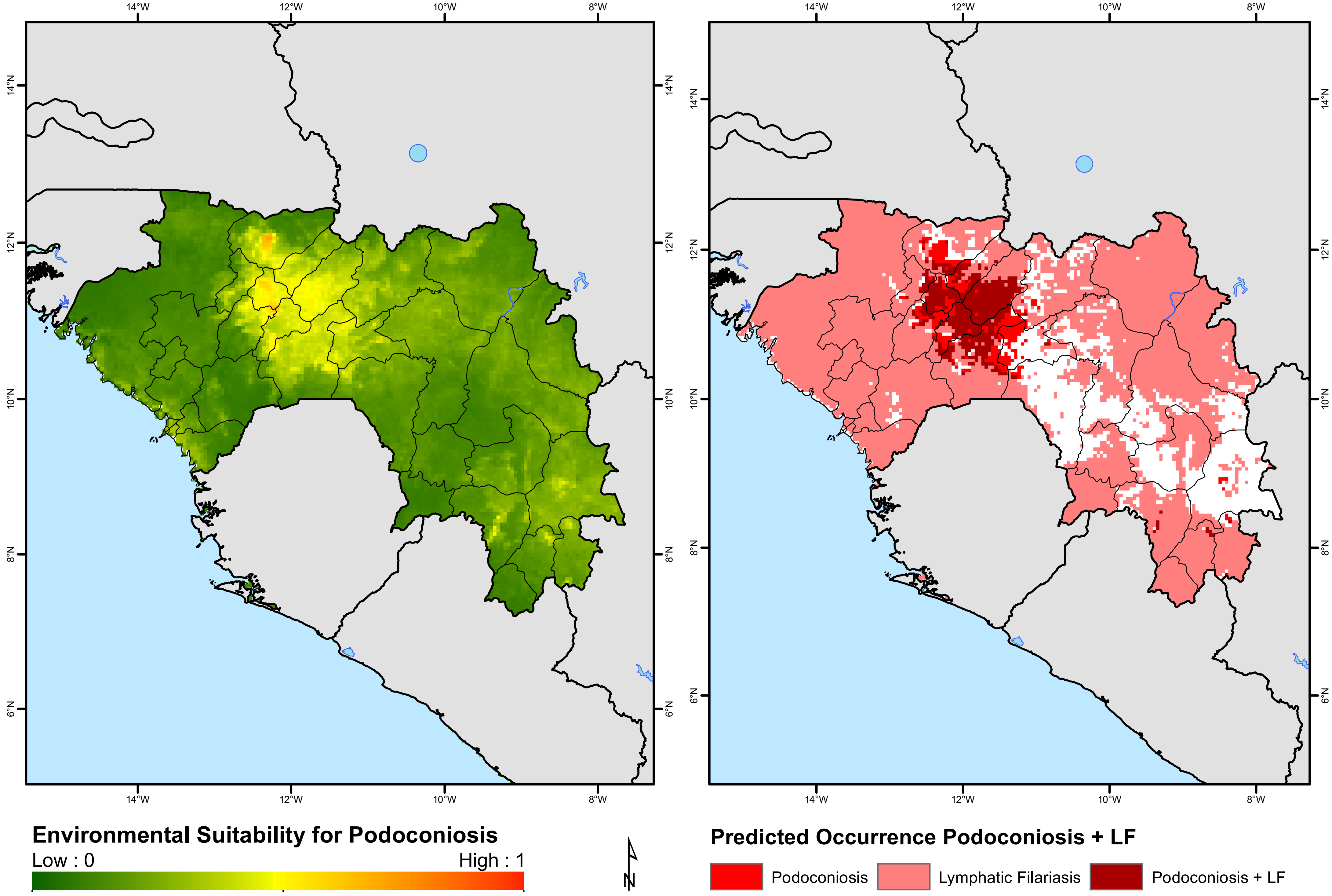
14°W
12°W
10°W
8°W
14°N
12°N
10°N
8°N
6°N
Environmental Suitability for Podoconiosis
Low : 0
High : 1
N
Predicted Occurrence Podoconiosis + LF
Podoconiosis
Lymphatic Filariasis
Podoconiosis + LF

Guinea-Bissau

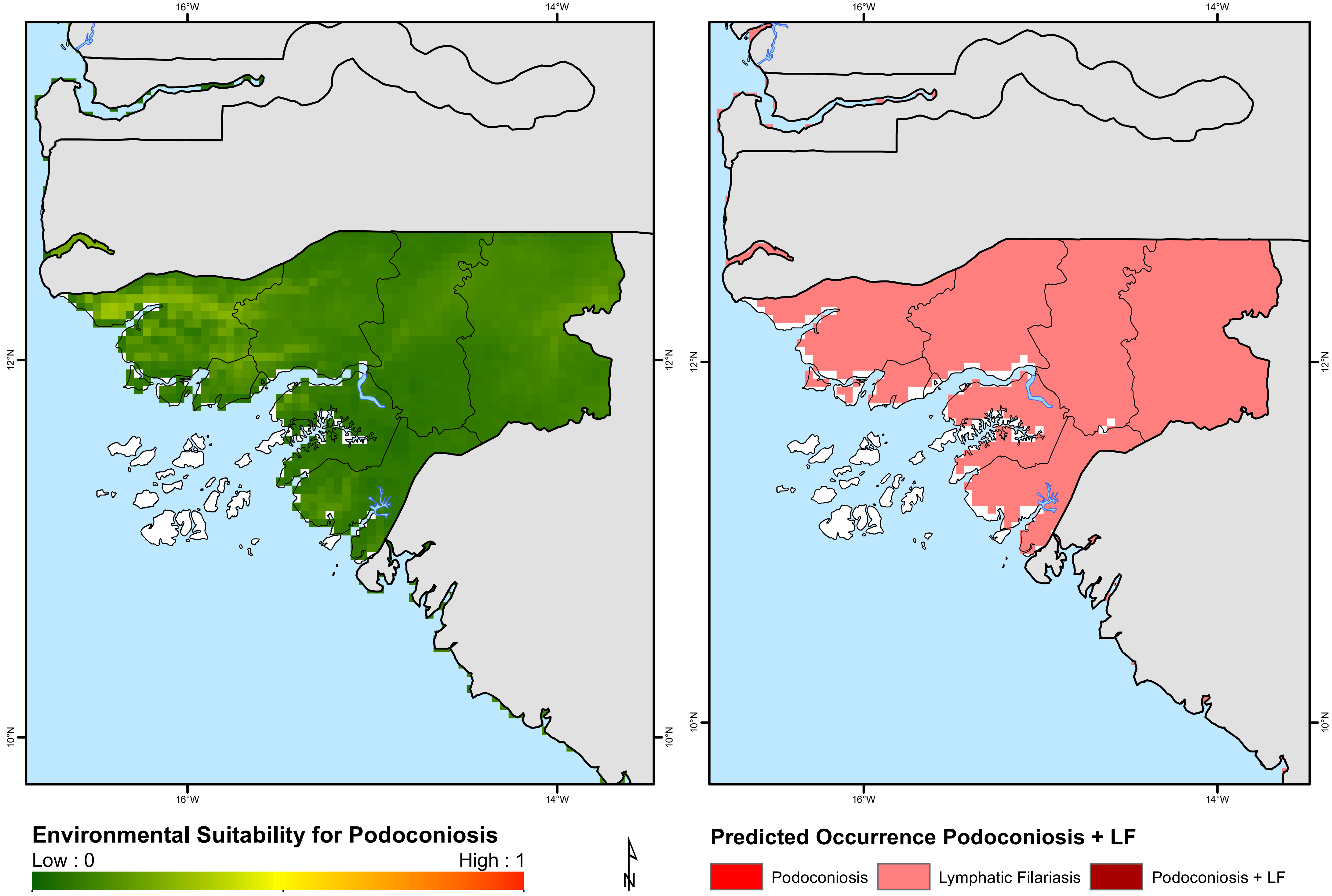

Kenya

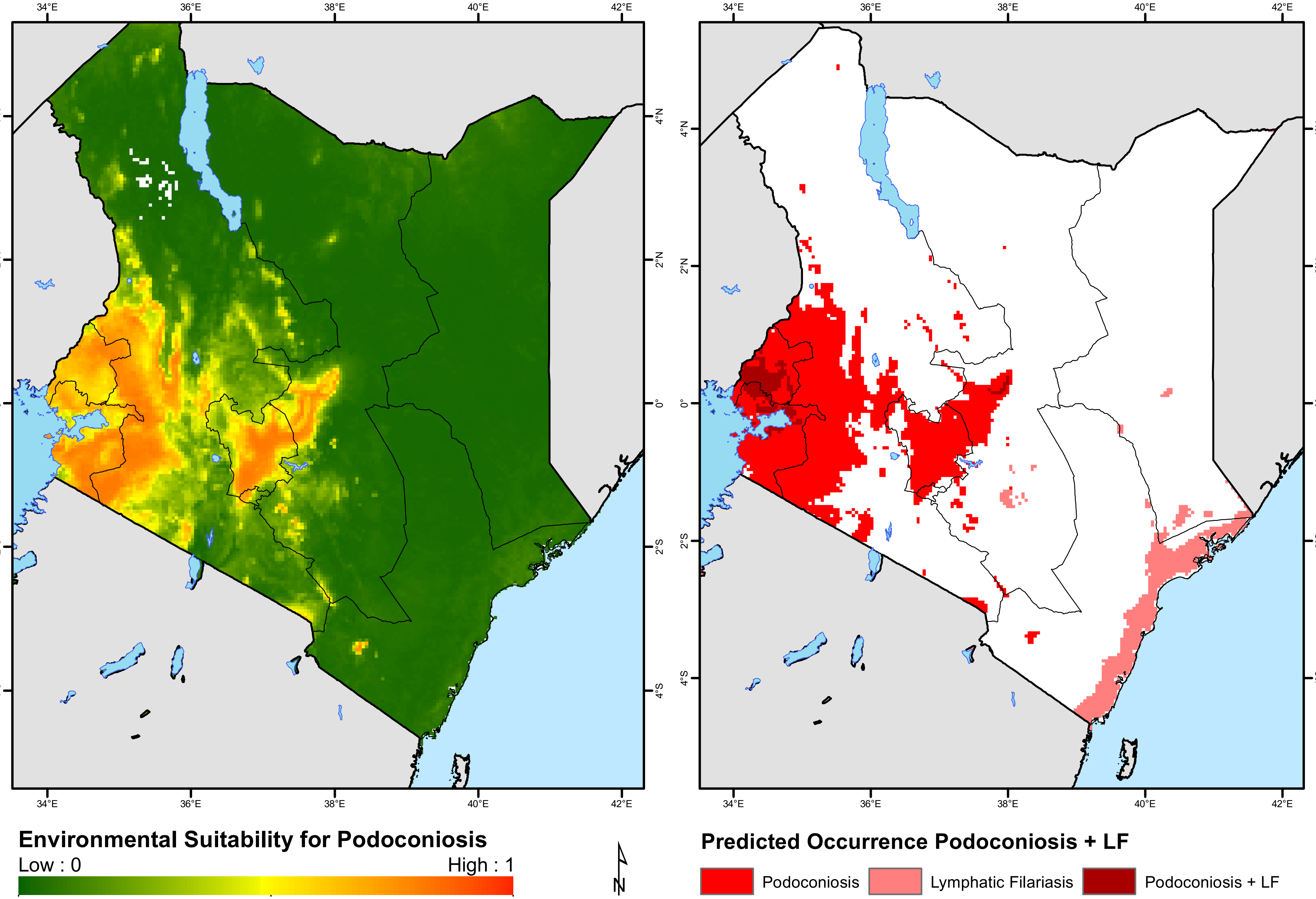
34°E
36°E
38°E
40°E
42°E
4°N
2°N
0°
2°S
4°S
Environmental Suitability for Podoconiosis
Low : 0
High : 1
N
Predicted Occurrence Podoconiosis + LF
Podoconiosis
Lymphatic Filariasis
Podoconiosis + LF

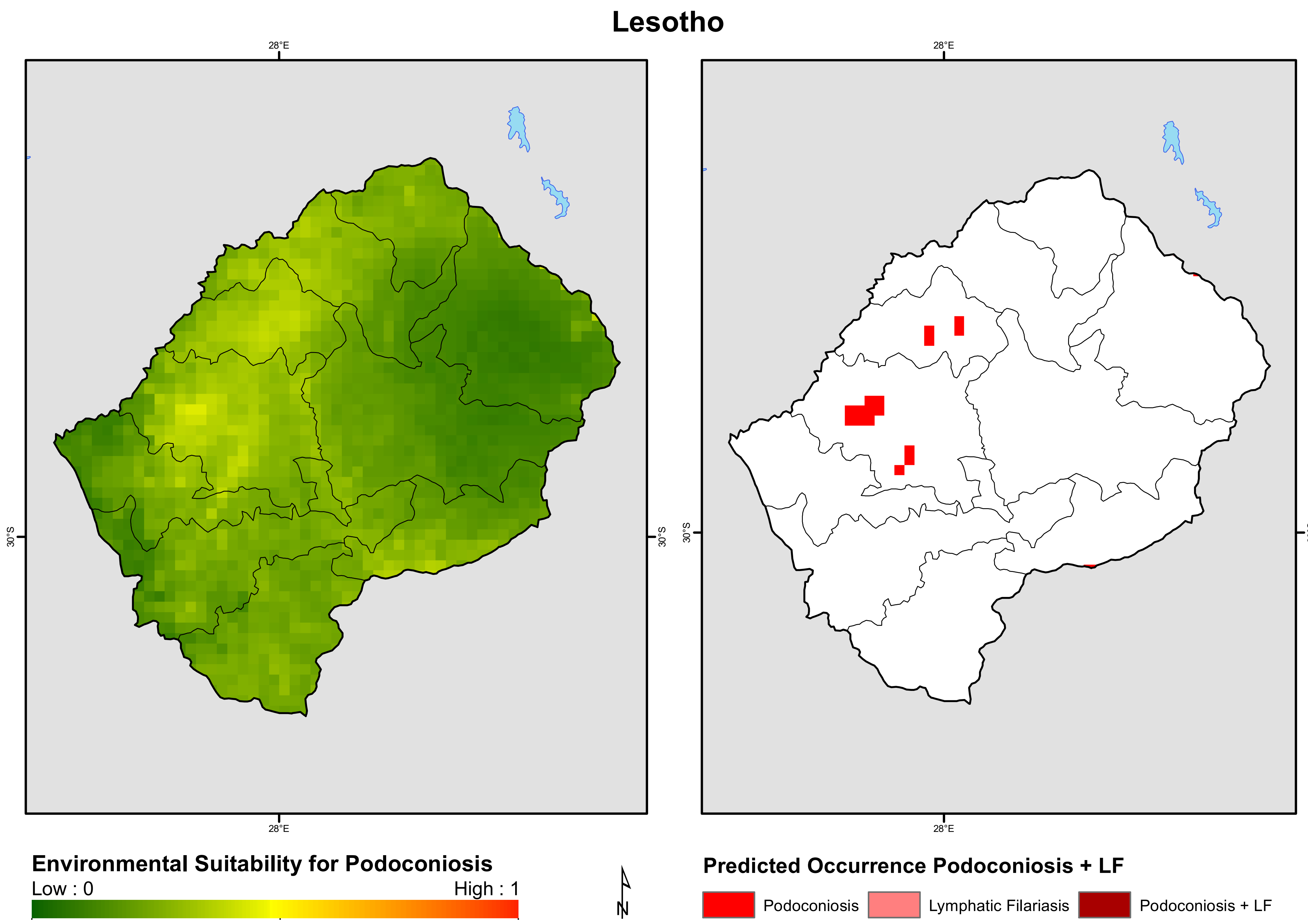

Lesotho
28°E
30°S
Environmental Suitability for Podoconiosis
Low : 0
High : 1
N
Predicted Occurrence Podoconiosis + LF
Podoconiosis
Lymphatic Filariasis
Podoconiosis + LF

Liberia

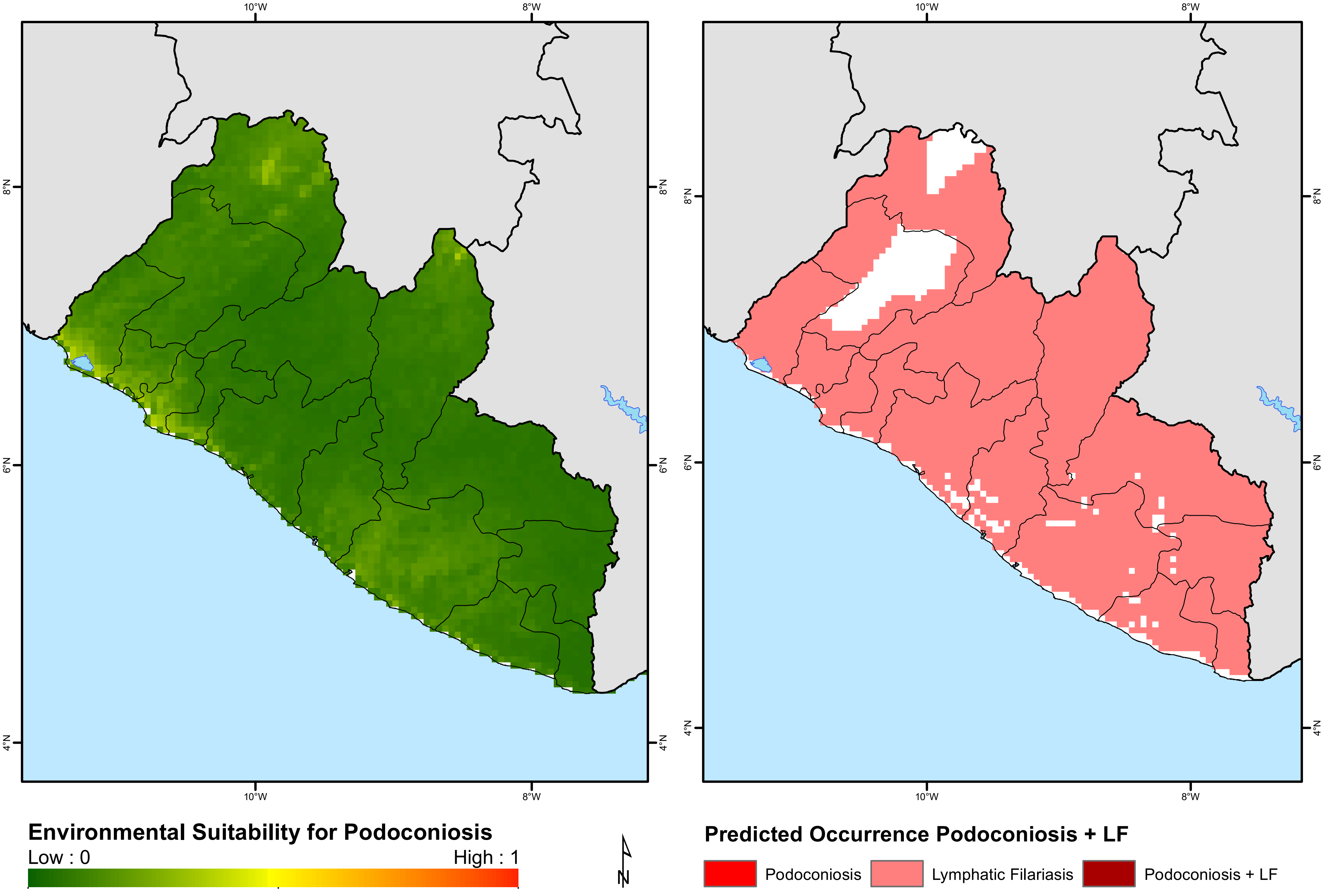
10°W
8°W
8°N
6°N
4°N
Environmental Suitability for Podoconiosis
Low : 0
High : 1
N
Predicted Occurrence Podoconiosis + LF
Podoconiosis
Lymphatic Filariasis
Podoconiosis + LF

Madagascar

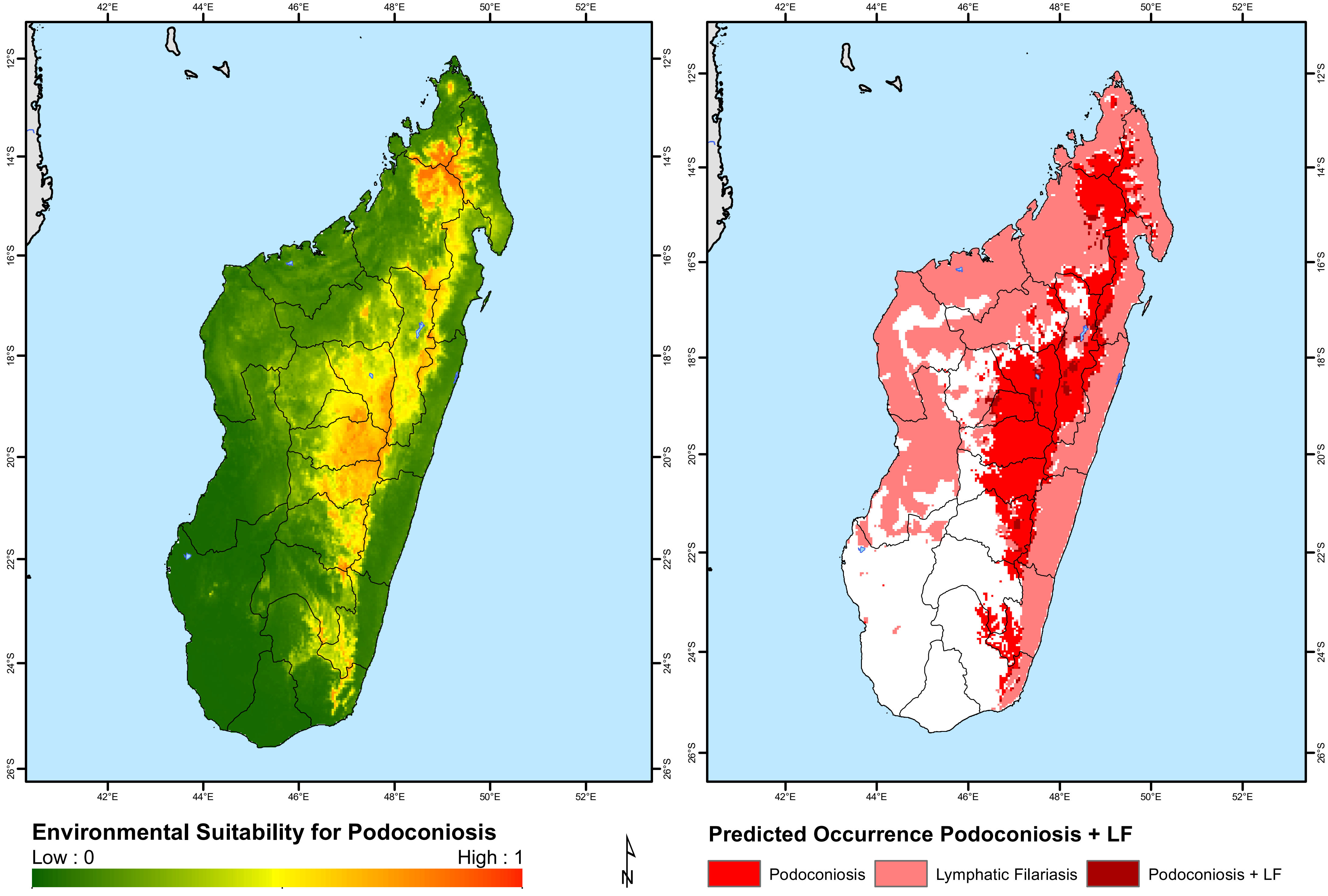
42°E
44°E
46°E
48°E
50°E
52°E
12°S
14°S
16°S
18°S
20°S
22°S
24°S
26°S
Environmental Suitability for Podoconiosis
Low : 0
High : 1
N
Predicted Occurrence Podoconiosis + LF
Podoconiosis
Lymphatic Filariasis
Podoconiosis + LF

Malawi

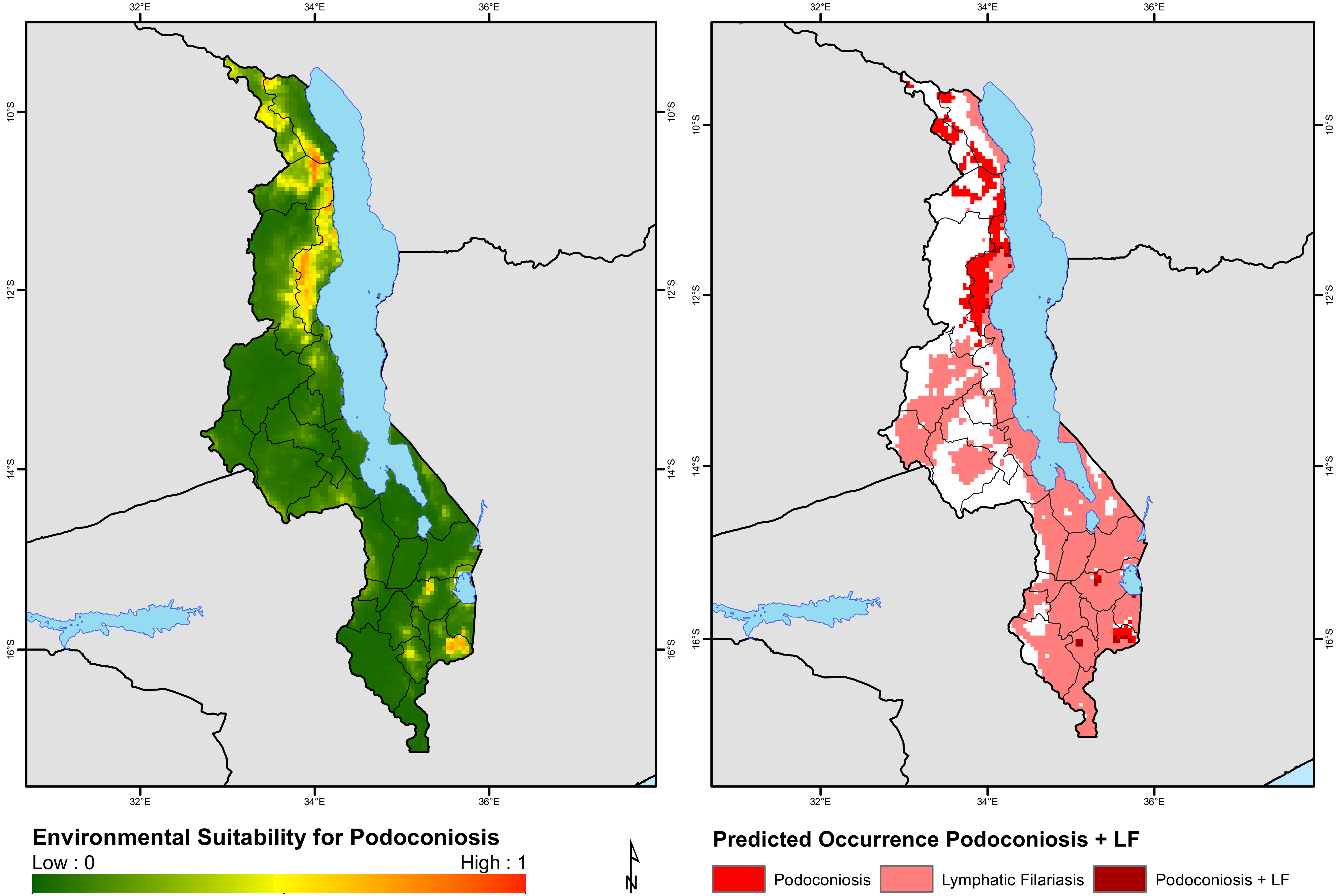
32°E
34°E
36°E
10°S
12°S
14°S
16°S
Environmental Suitability for Podoconiosis
Low : 0
High : 1
N
Predicted Occurrence Podoconiosis + LF
Podoconiosis
Lymphatic Filariasis
Podoconiosis + LF

Mali

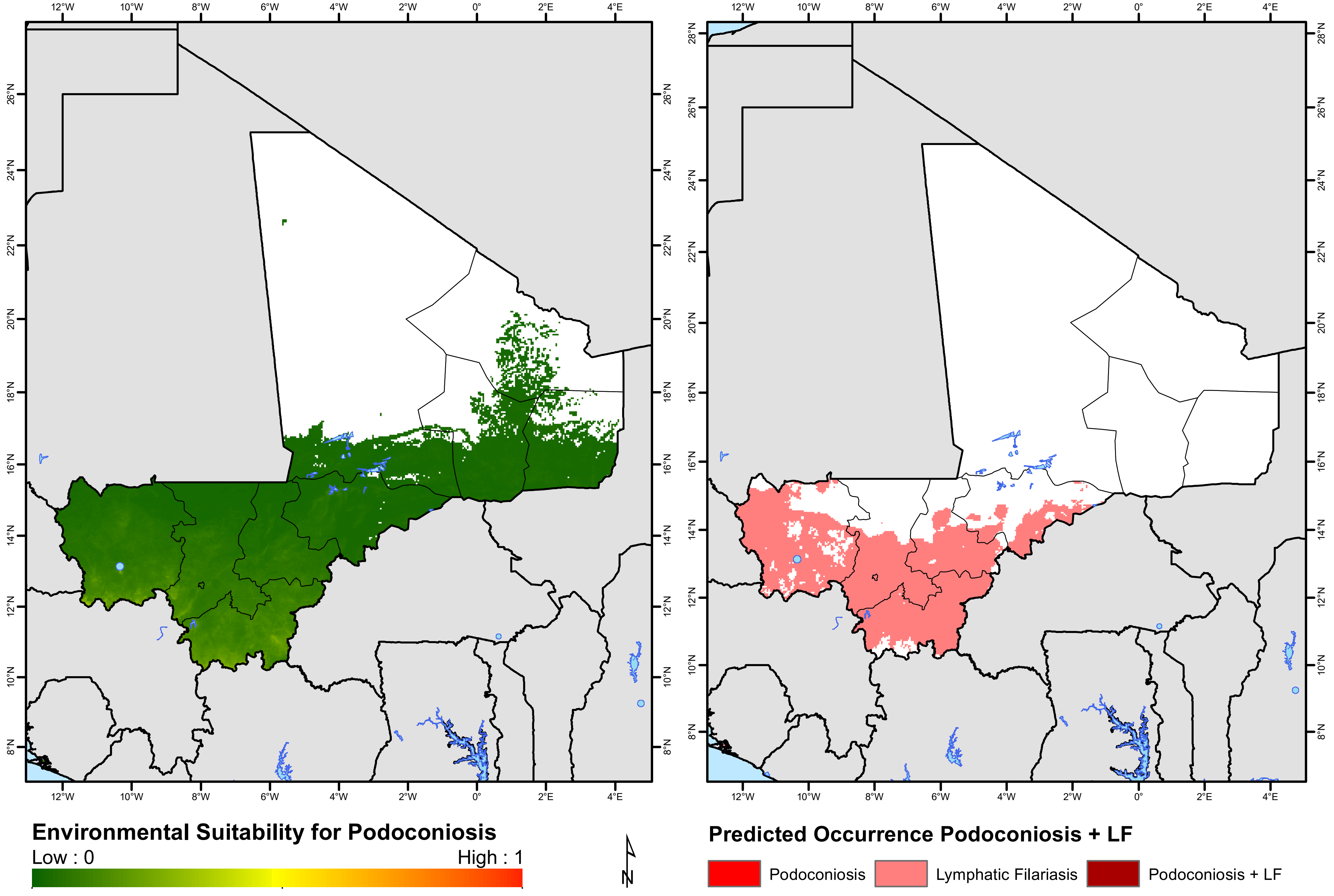

12°W
10°W
8°W
6°W
4°W
2°W
0°
2°E
4°E
28°N
26°N
24°N
22°N
20°N
18°N
16°N
14°N
12°N
10°N
8°N
Environmental Suitability for Podoconiosis
Low : 0
High : 1
N
Predicted Occurrence Podoconiosis + LF
Podoconiosis
Lymphatic Filariasis
Podoconiosis + LF

Mauritania

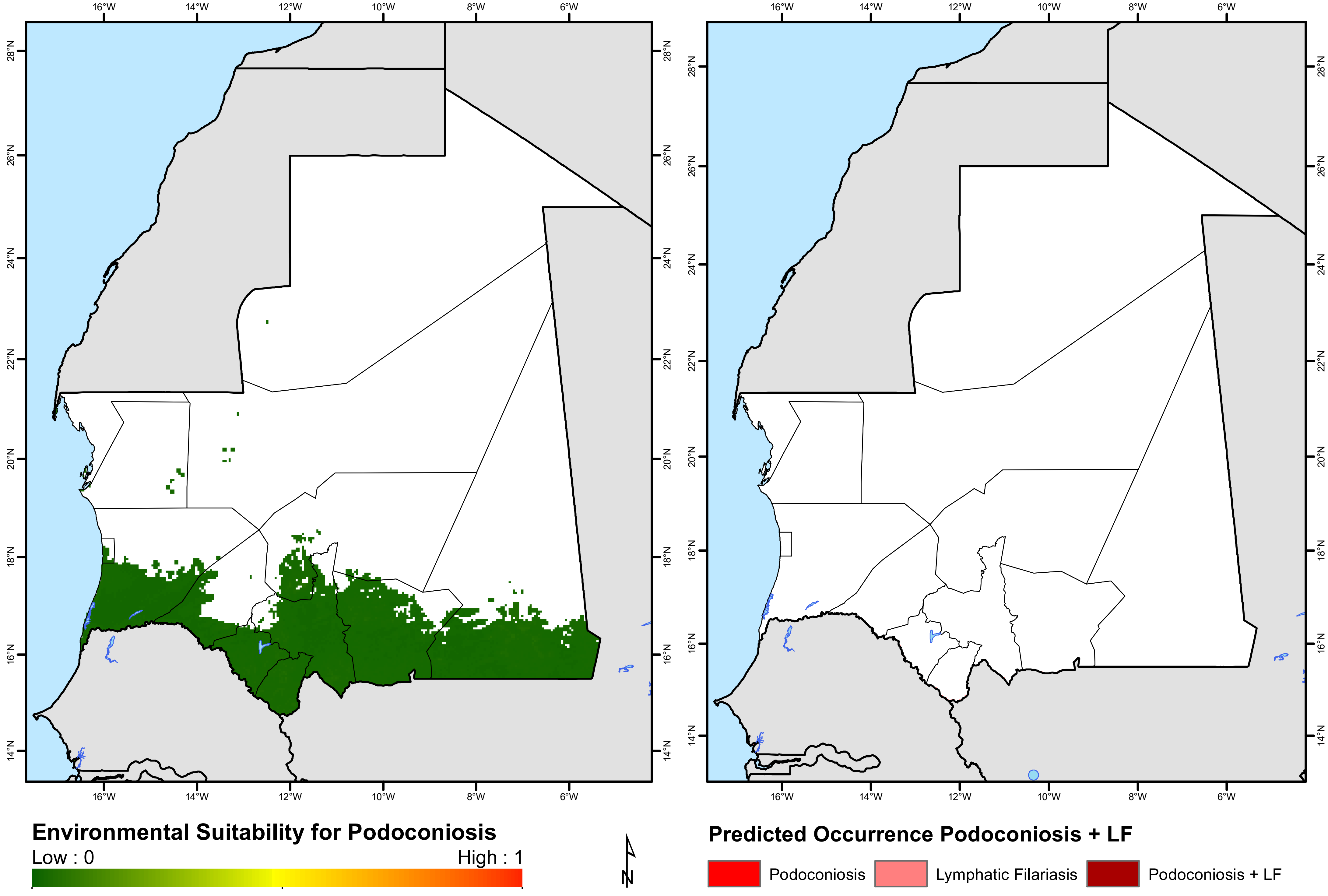

Environmental Suitability for Podoconiosis
Low : 0
High : 1
N
Predicted Occurrence Podoconiosis + LF
Podoconiosis
Lymphatic Filariasis
Podoconiosis + LF
16°W
14°W
12°W
10°W
8°W
6°W
28°N
26°N
24°N
22°N
20°N
18°N
16°N
14°N

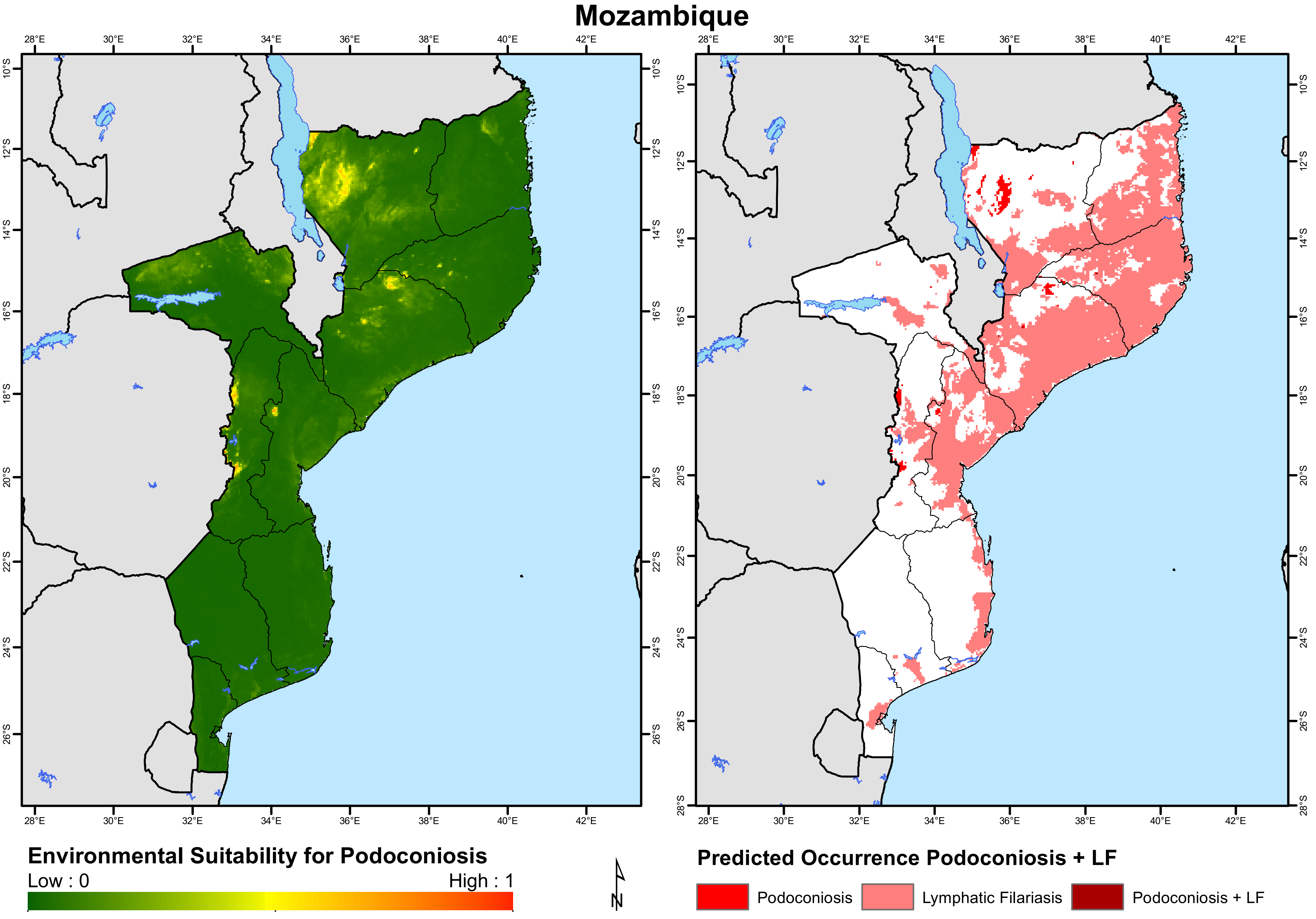

Mozambique
28°E
30°E
32°E
34°E
36°E
38°E
40°E
42°E
10°S
12°S
14°S
16°S
18°S
20°S
22°S
24°S
26°S
28°S
Environmental Suitability for Podoconiosis
Low : 0
High : 1
N
Predicted Occurrence Podoconiosis + LF
Podoconiosis
Lymphatic Filariasis
Podoconiosis + LF

Namibia

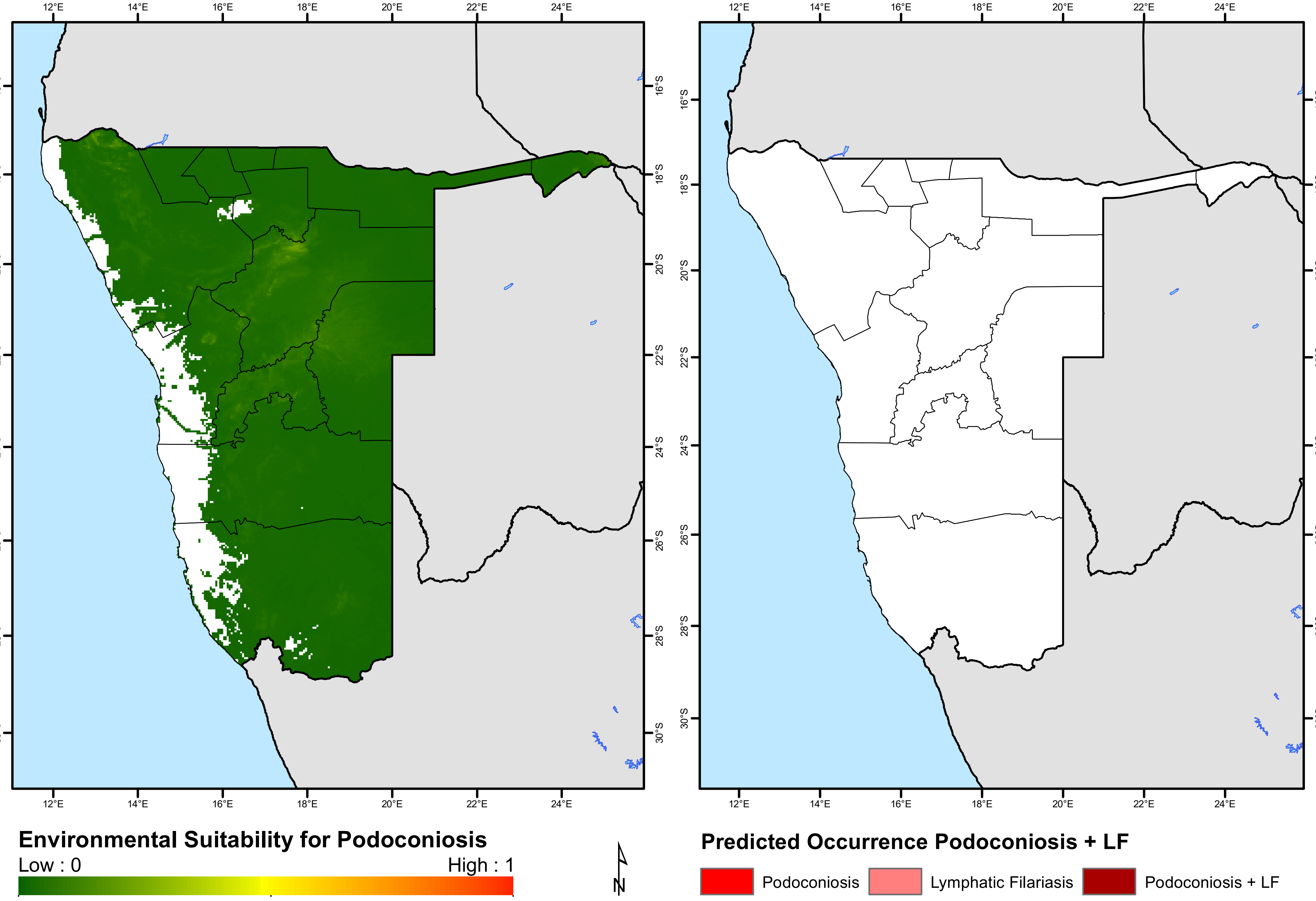

12°E
14°E
16°E
18°E
20°E
22°E
24°E
16°S
18°S
20°S
22°S
24°S
26°S
28°S
30°S
Environmental Suitability for Podoconiosis
Low : 0
High : 1
N
Predicted Occurrence Podoconiosis + LF
Podoconiosis
Lymphatic Filariasis
Podoconiosis + LF

Niger

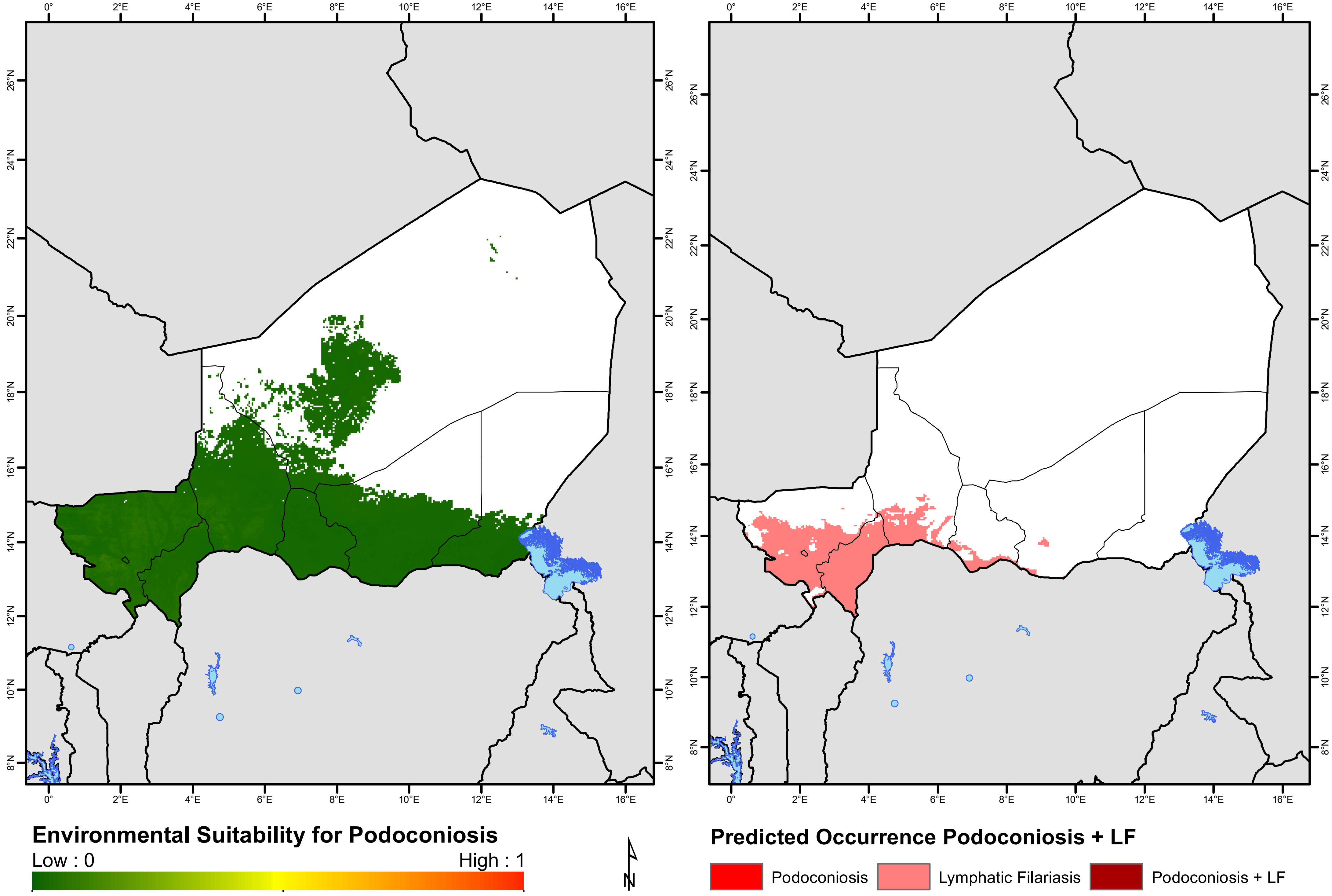
0°
2°E
4°E
6°E
8°E
10°E
12°E
14°E
16°E
8°N
10°N
12°N
14°N
16°N
18°N
20°N
22°N
24°N
26°N
Environmental Suitability for Podoconiosis
Low : 0
High : 1
N
Predicted Occurrence Podoconiosis + LF
Podoconiosis
Lymphatic Filariasis
Podoconiosis + LF

Nigeria

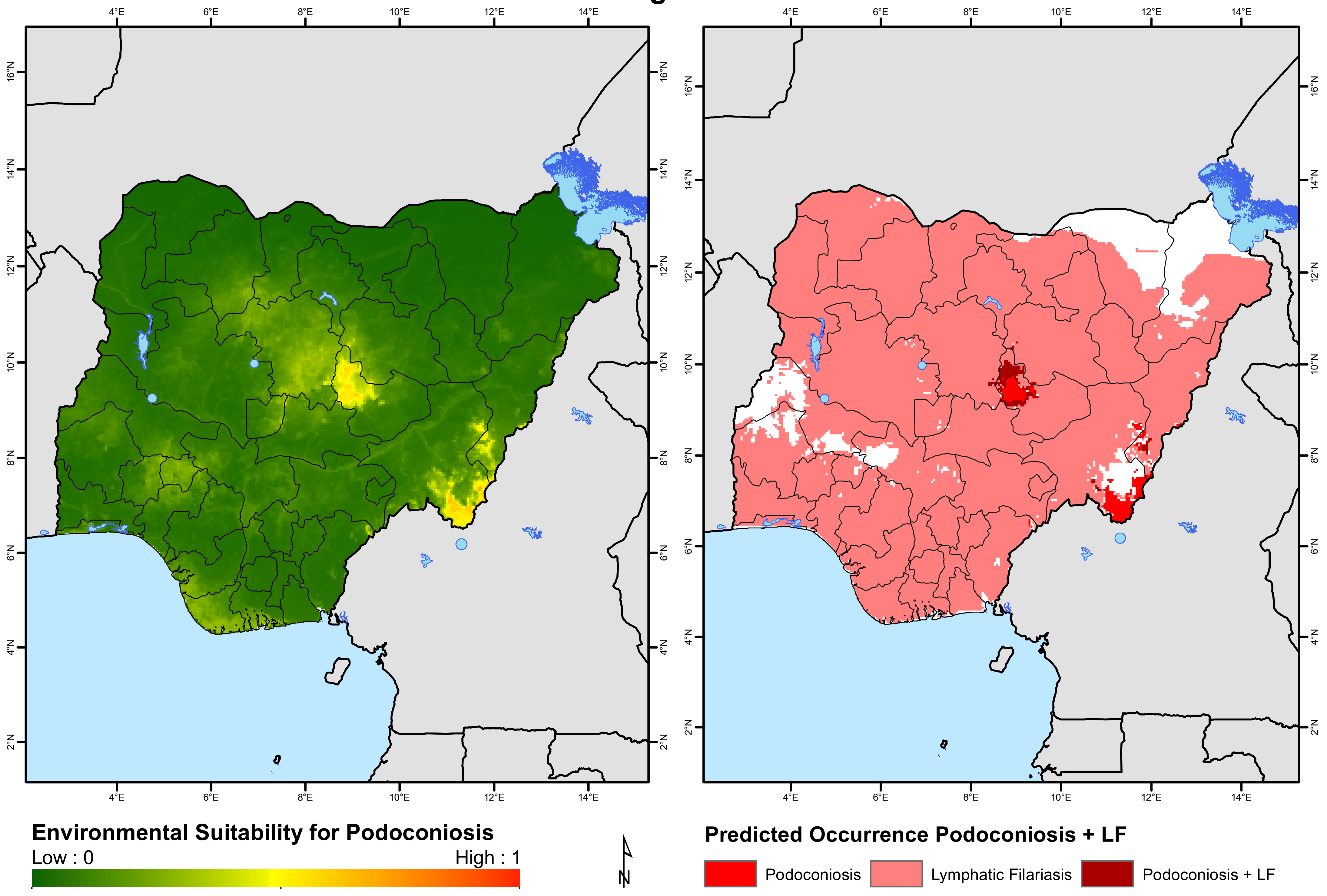
4°E
6°E
8°E
10°E
12°E
14°E
16°N
14°N
12°N
10°N
8°N
6°N
4°N
2°N
Environmental Suitability for Podoconiosis
Low : 0
High : 1
N
Predicted Occurrence Podoconiosis + LF
Podoconiosis
Lymphatic Filariasis
Podoconiosis + LF

Rwanda

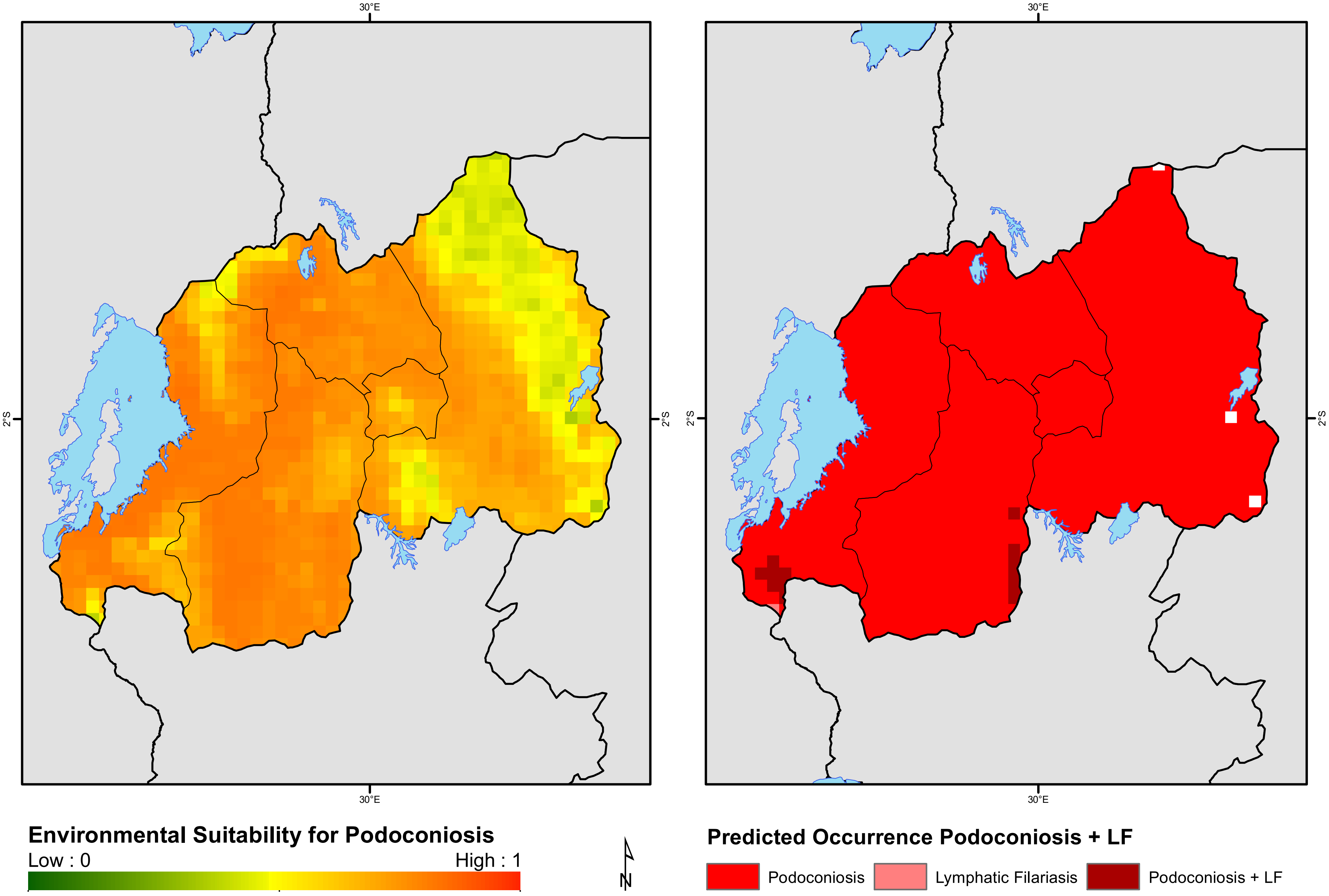
30°E
2°S
Environmental Suitability for Podoconiosis
Low : 0
High : 1
N
Predicted Occurrence Podoconiosis + LF
Podoconiosis
Lymphatic Filariasis
Podoconiosis + LF

Sao Tome and Principe

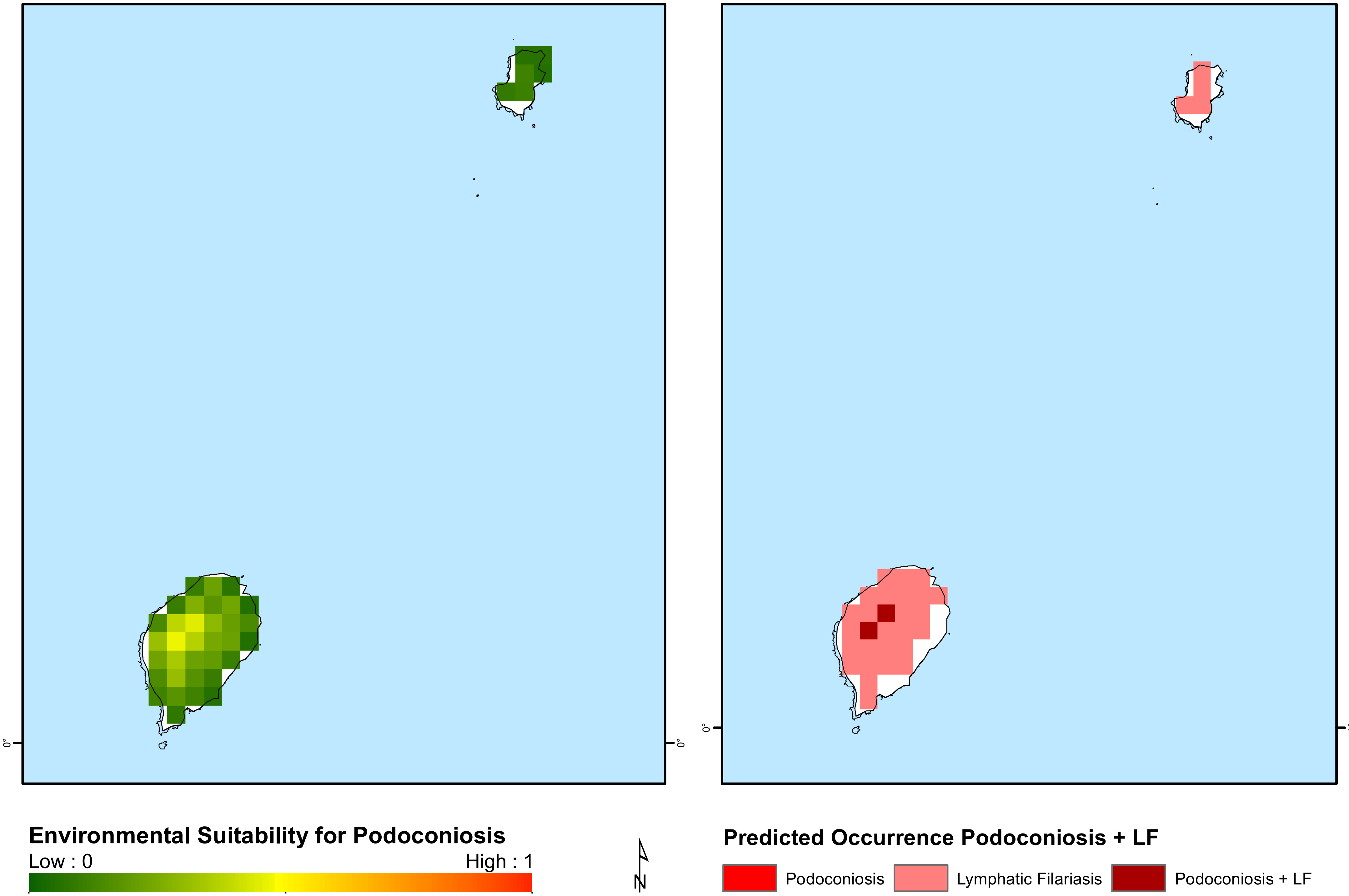

Senegal

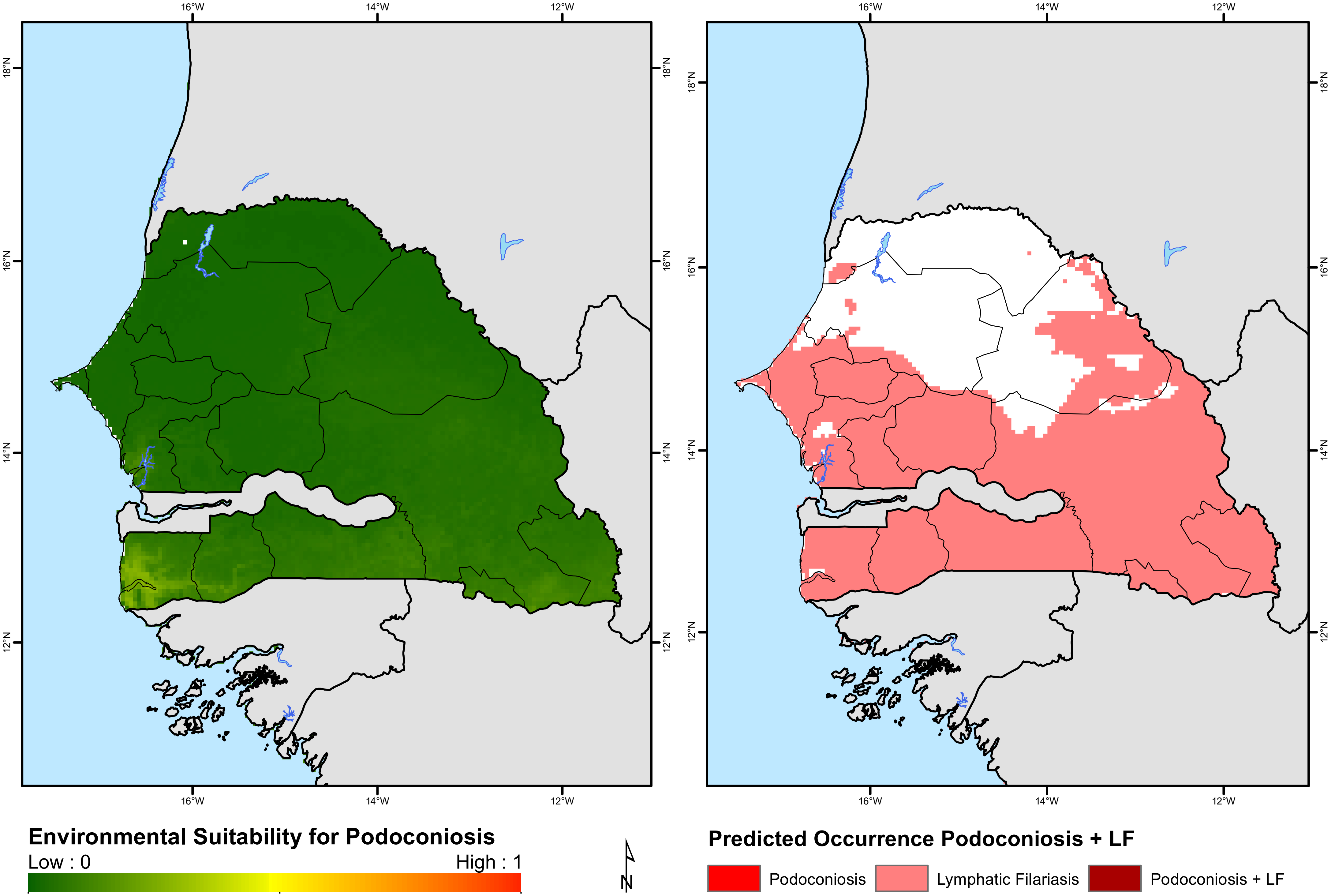
16°W
14°W
12°W
18°N
16°N
14°N
12°N
Environmental Suitability for Podoconiosis
Low : 0
High : 1
N
Predicted Occurrence Podoconiosis + LF
Podoconiosis
Lymphatic Filariasis
Podoconiosis + LF

Sierra Leone

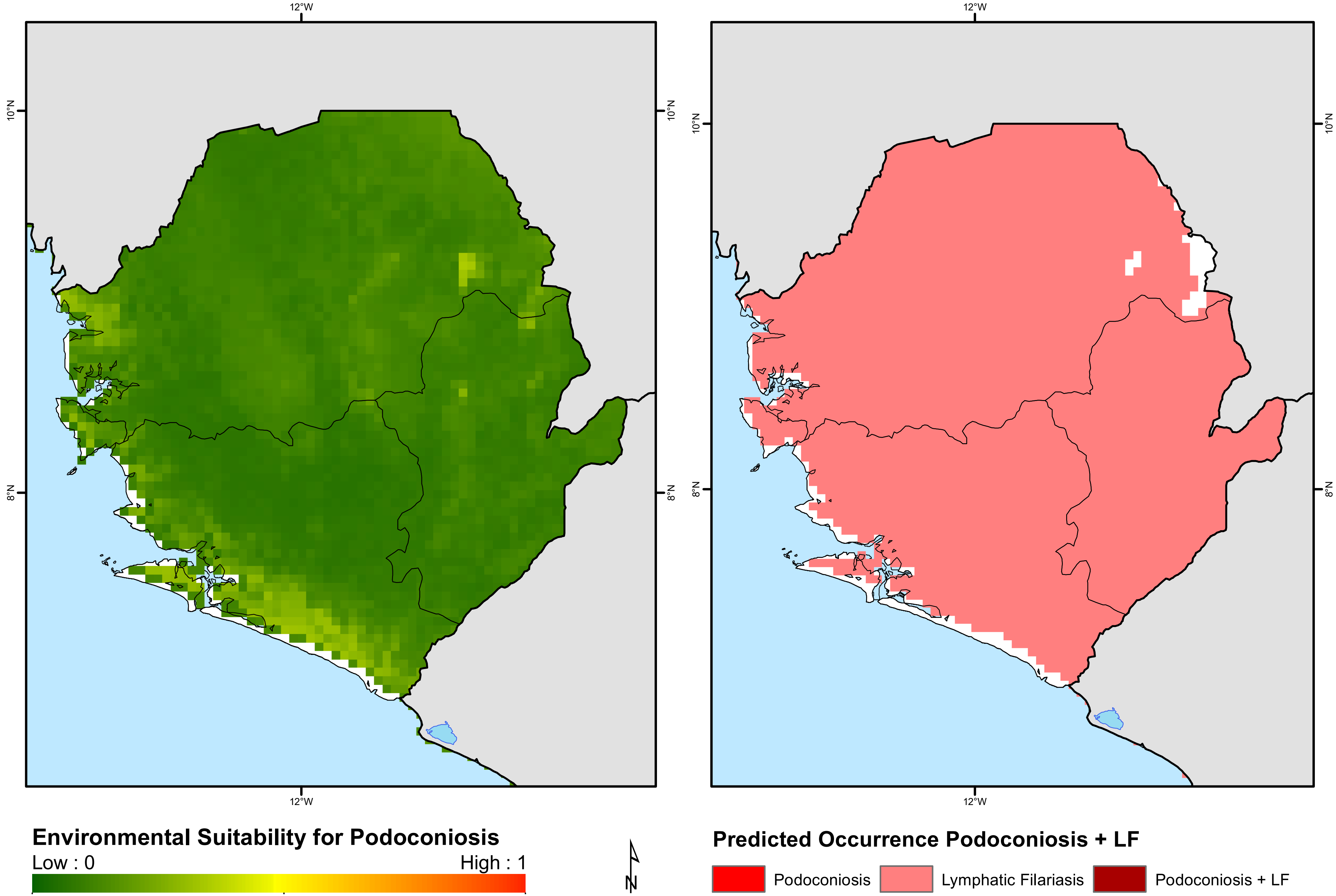

Environmental Suitability for Podoconiosis

Low : 0 High : 1

Predicted Occurrence Podoconiosis + LF

Podoconiosis | Lymphatic Filariasis | Podoconiosis + LF

Somalia

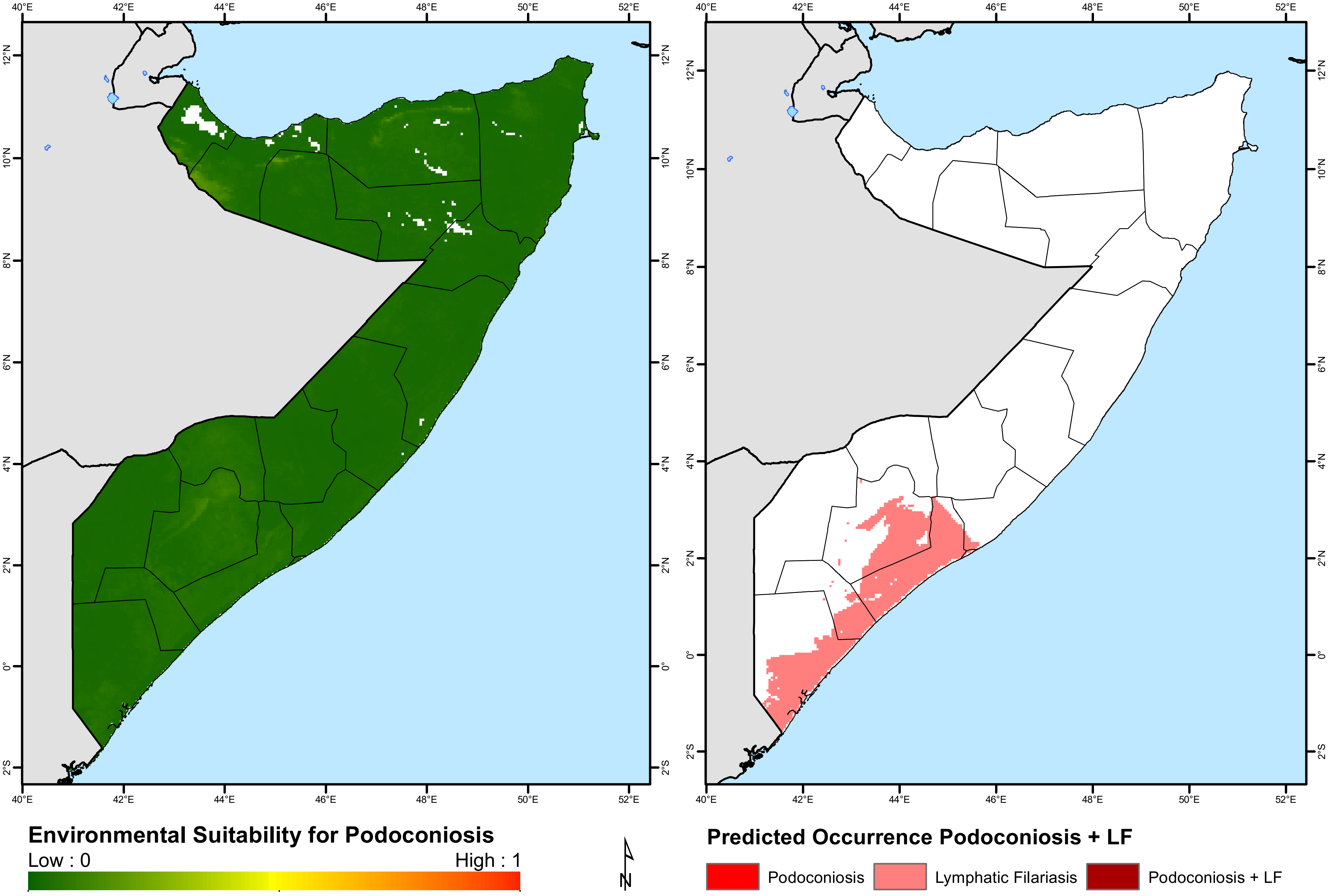
40°E
42°E
44°E
46°E
48°E
50°E
52°E
12°N
10°N
8°N
6°N
4°N
2°N
0°
2°S
Environmental Suitability for Podoconiosis
Low : 0
High : 1
N
Predicted Occurrence Podoconiosis + LF
Podoconiosis
Lymphatic Filariasis
Podoconiosis + LF

South Africa

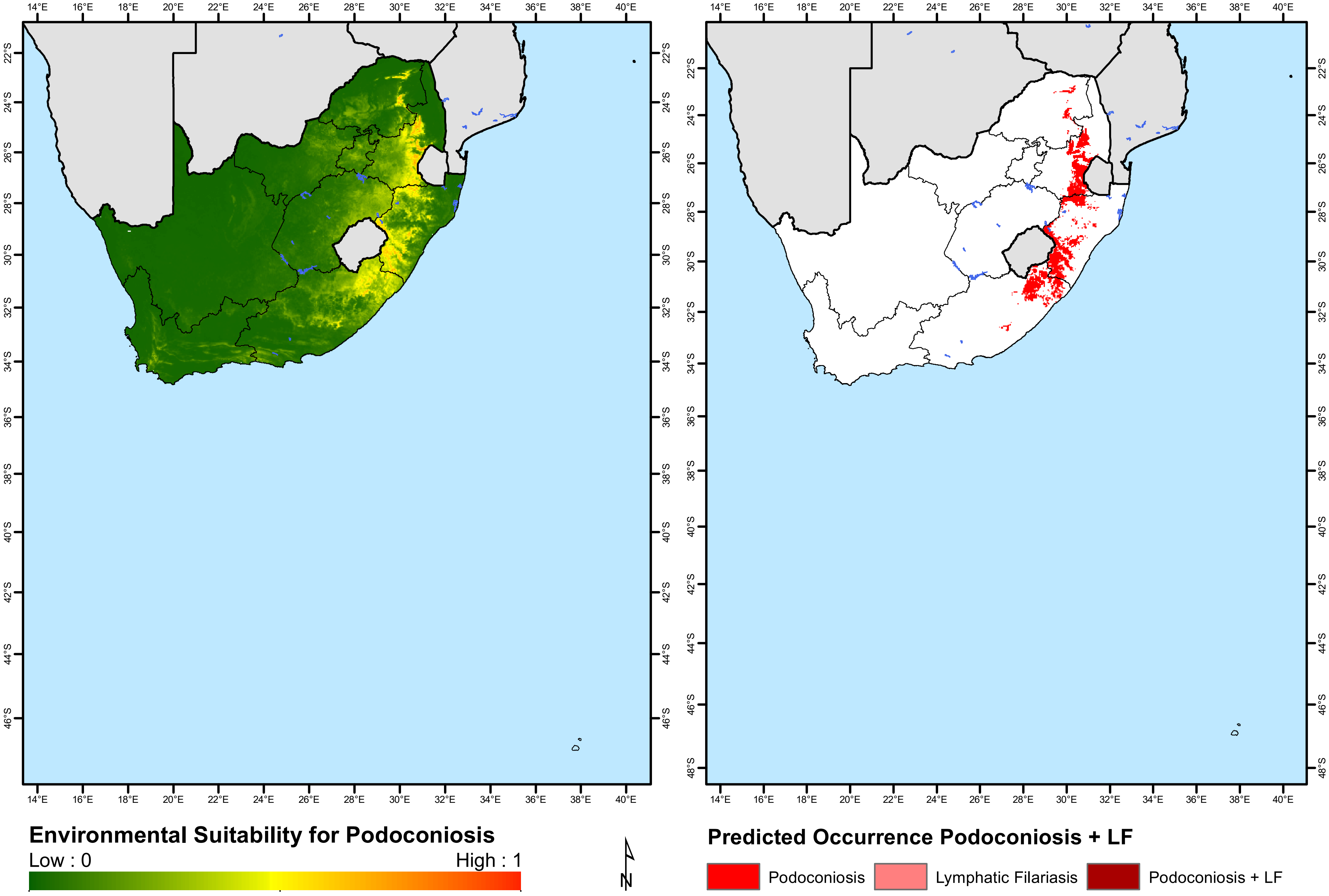

South Sudan

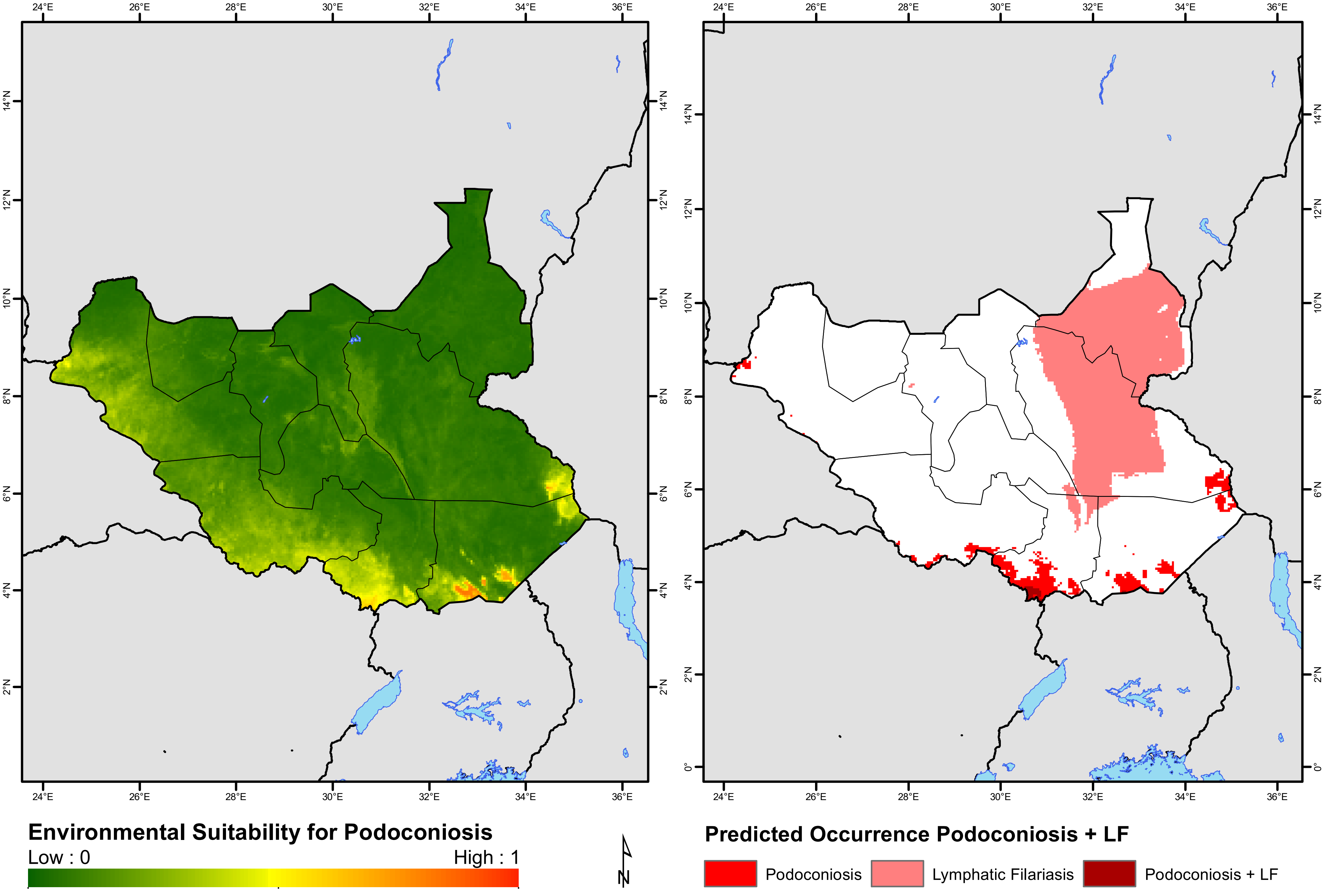
Environmental Suitability for Podoconiosis
Low : 0
High : 1
N
Predicted Occurrence Podoconiosis + LF
Podoconiosis
Lymphatic Filariasis
Podoconiosis + LF

Sudan

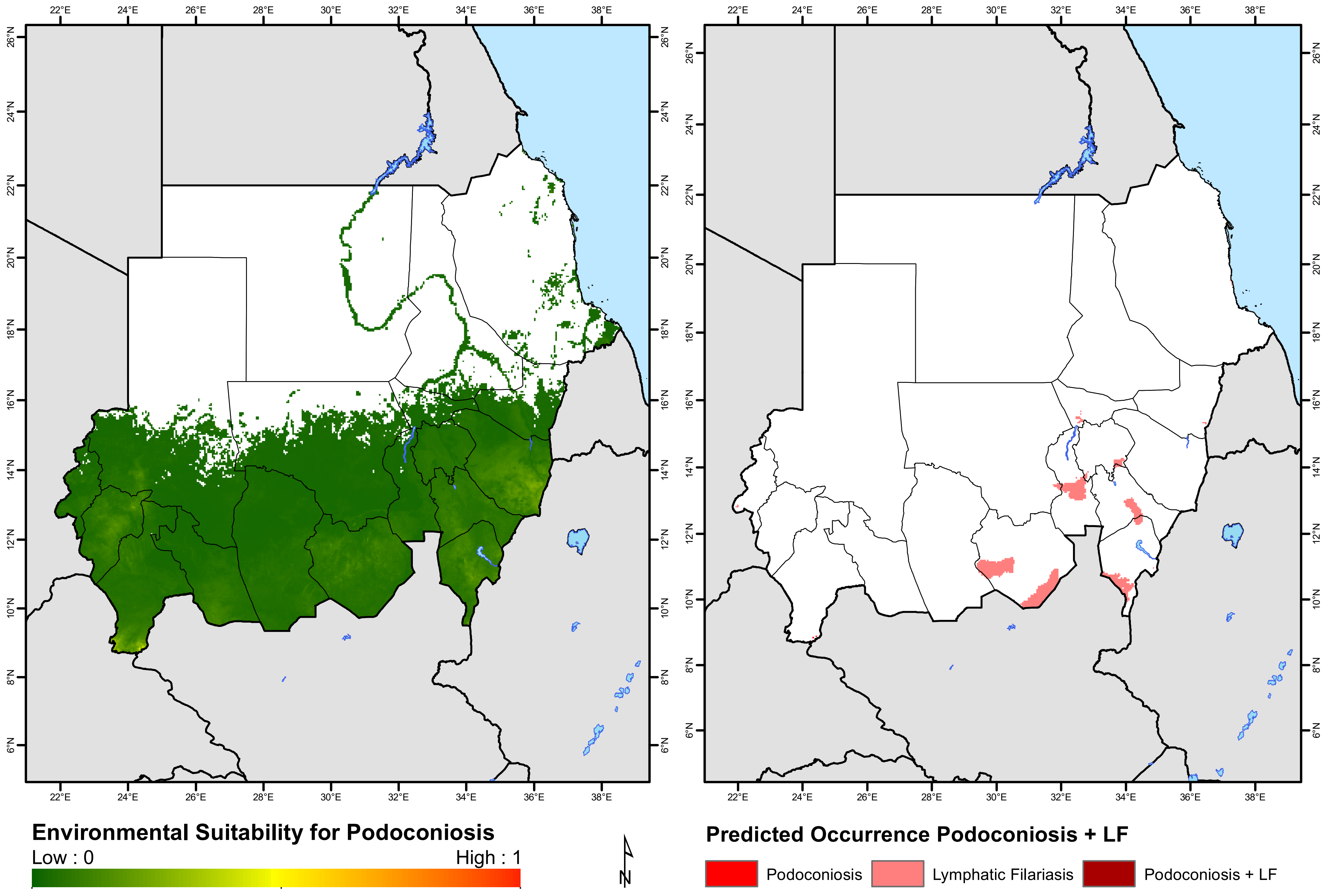
22°E
24°E
26°E
28°E
30°E
32°E
34°E
36°E
38°E
26°N
24°N
22°N
20°N
18°N
16°N
14°N
12°N
10°N
8°N
6°N
Environmental Suitability for Podoconiosis
Low : 0
High : 1
N
Predicted Occurrence Podoconiosis + LF
Podoconiosis
Lymphatic Filariasis
Podoconiosis + LF

Swaziland

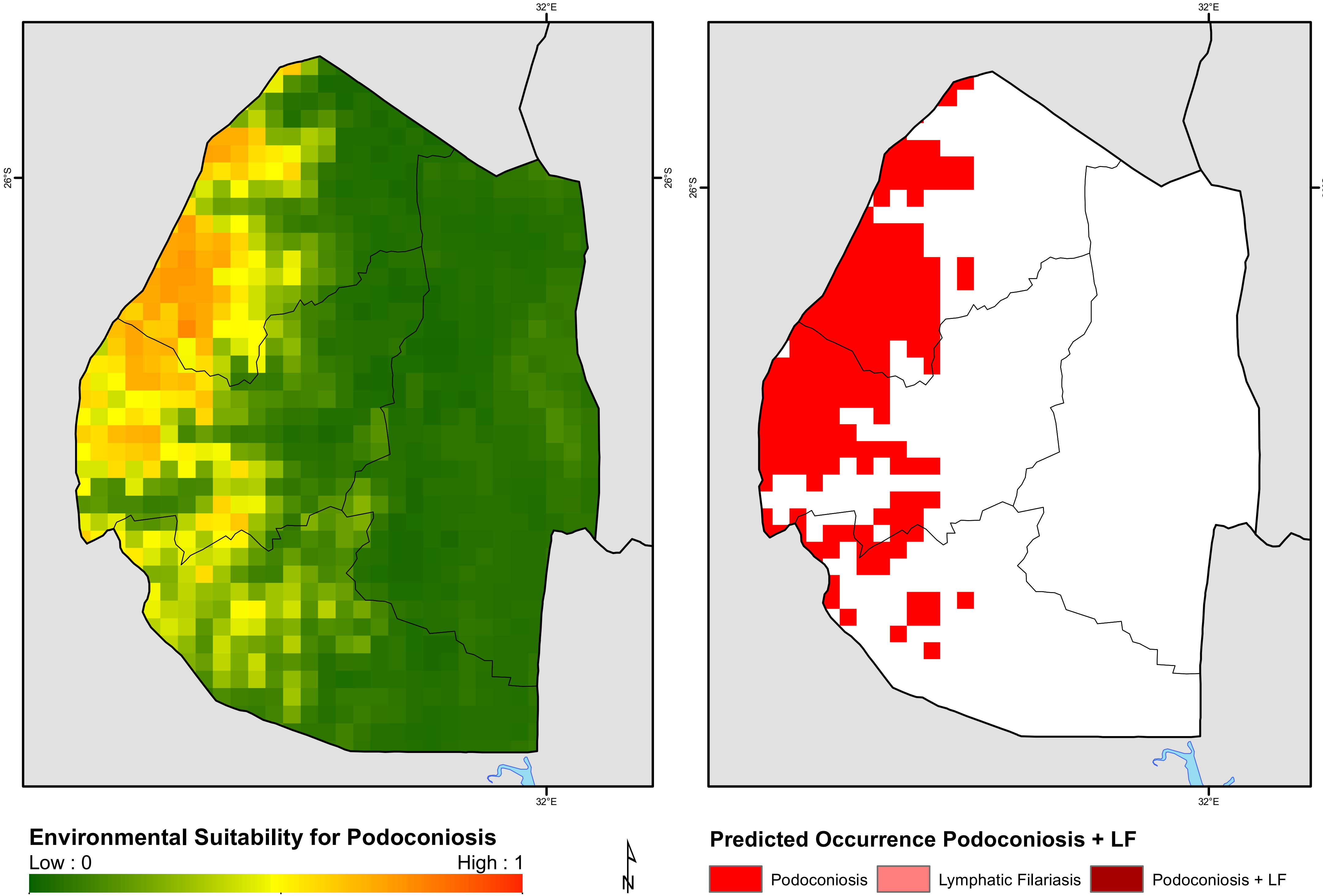
32°E
26°S
Environmental Suitability for Podoconiosis
Low : 0
High : 1
N
Predicted Occurrence Podoconiosis + LF
Podoconiosis
Lymphatic Filariasis
Podoconiosis + LF

Togo

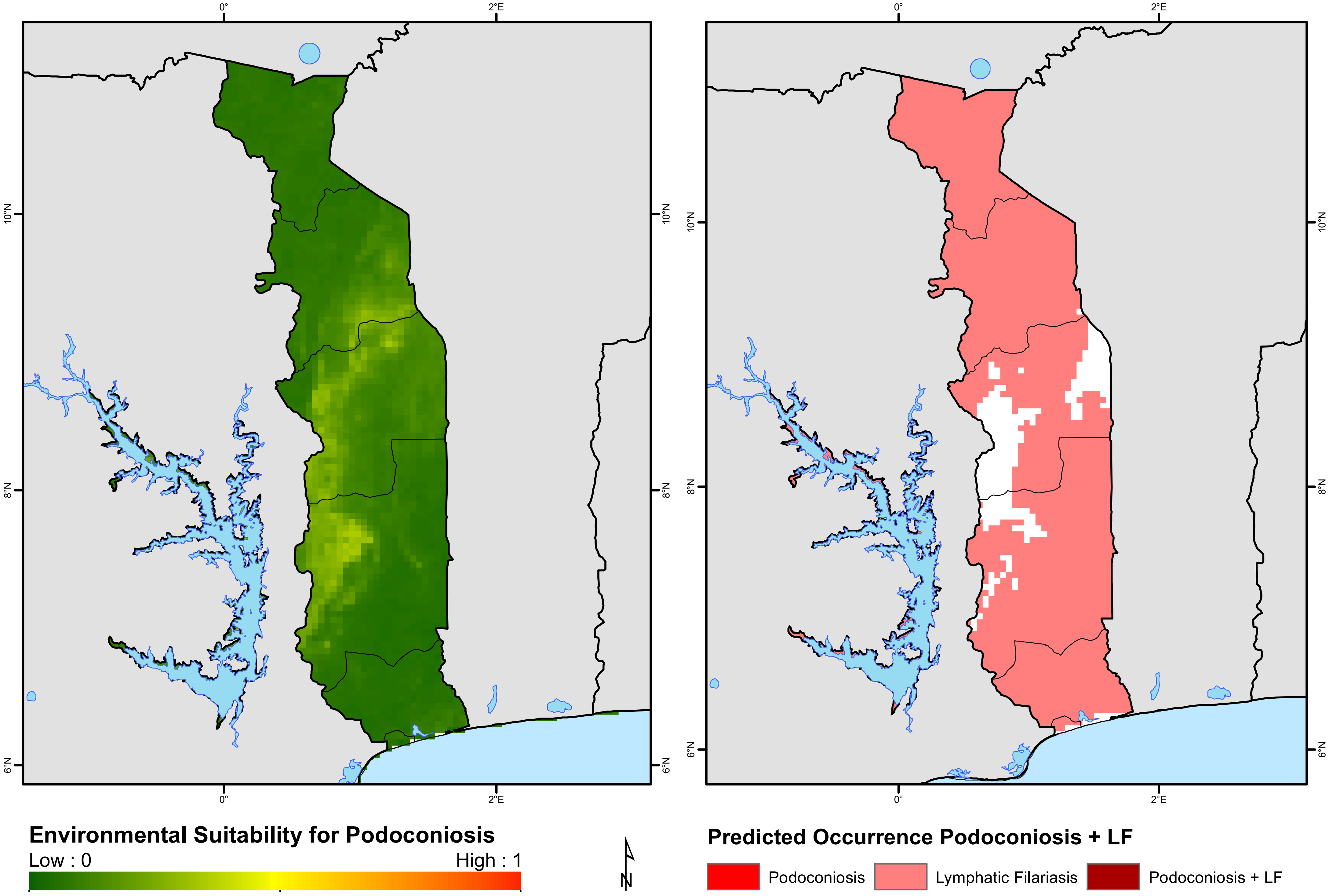

0°
2°E
10°N
8°N
6°N
Environmental Suitability for Podoconiosis
Low : 0
High : 1
N
Predicted Occurrence Podoconiosis + LF
Podoconiosis
Lymphatic Filariasis
Podoconiosis + LF

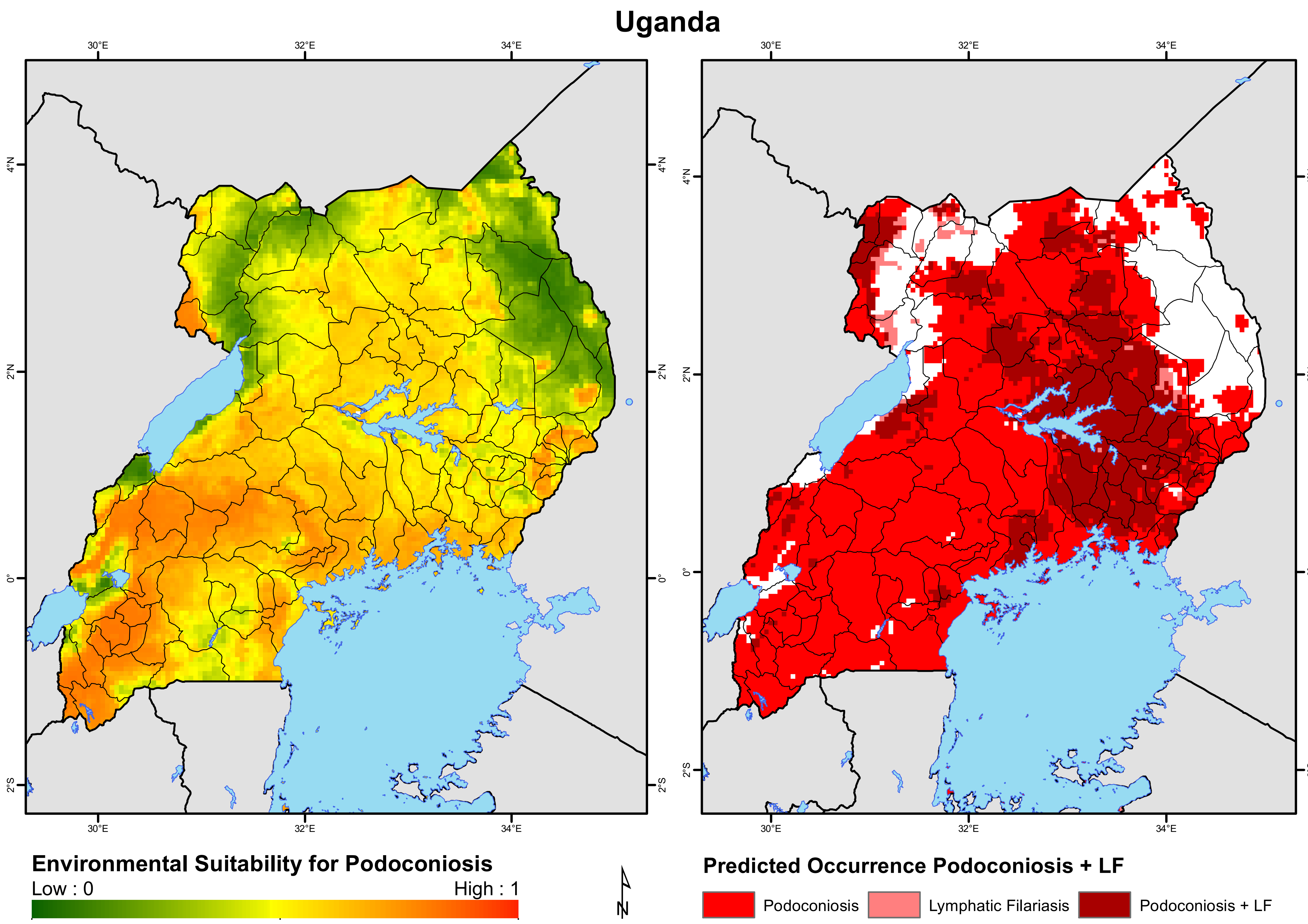
Uganda
30°E
32°E
34°E
4°N
2°N
0°
2°S
Environmental Suitability for Podoconiosis
Low : 0
High : 1
Predicted Occurrence Podoconiosis + LF
Podoconiosis
Lymphatic Filariasis
Podoconiosis + LF
N

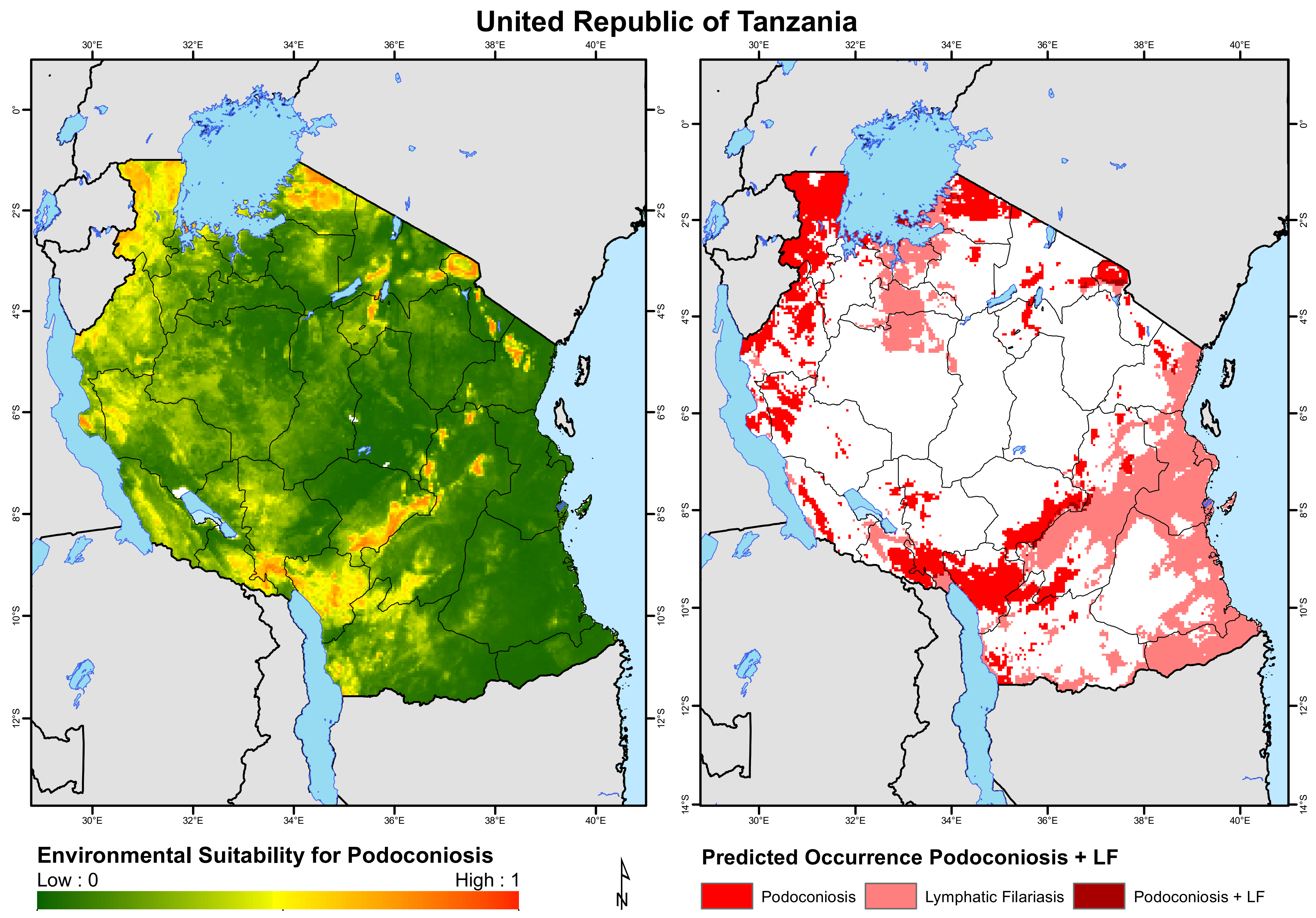
United Republic of Tanzania
30°E
32°E
34°E
36°E
38°E
40°E
0°
2°S
4°S
6°S
8°S
10°S
12°S
14°S
Environmental Suitability for Podoconiosis
Low : 0
High : 1
N
Predicted Occurrence Podoconiosis + LF
Podoconiosis
Lymphatic Filariasis
Podoconiosis + LF

Zambia

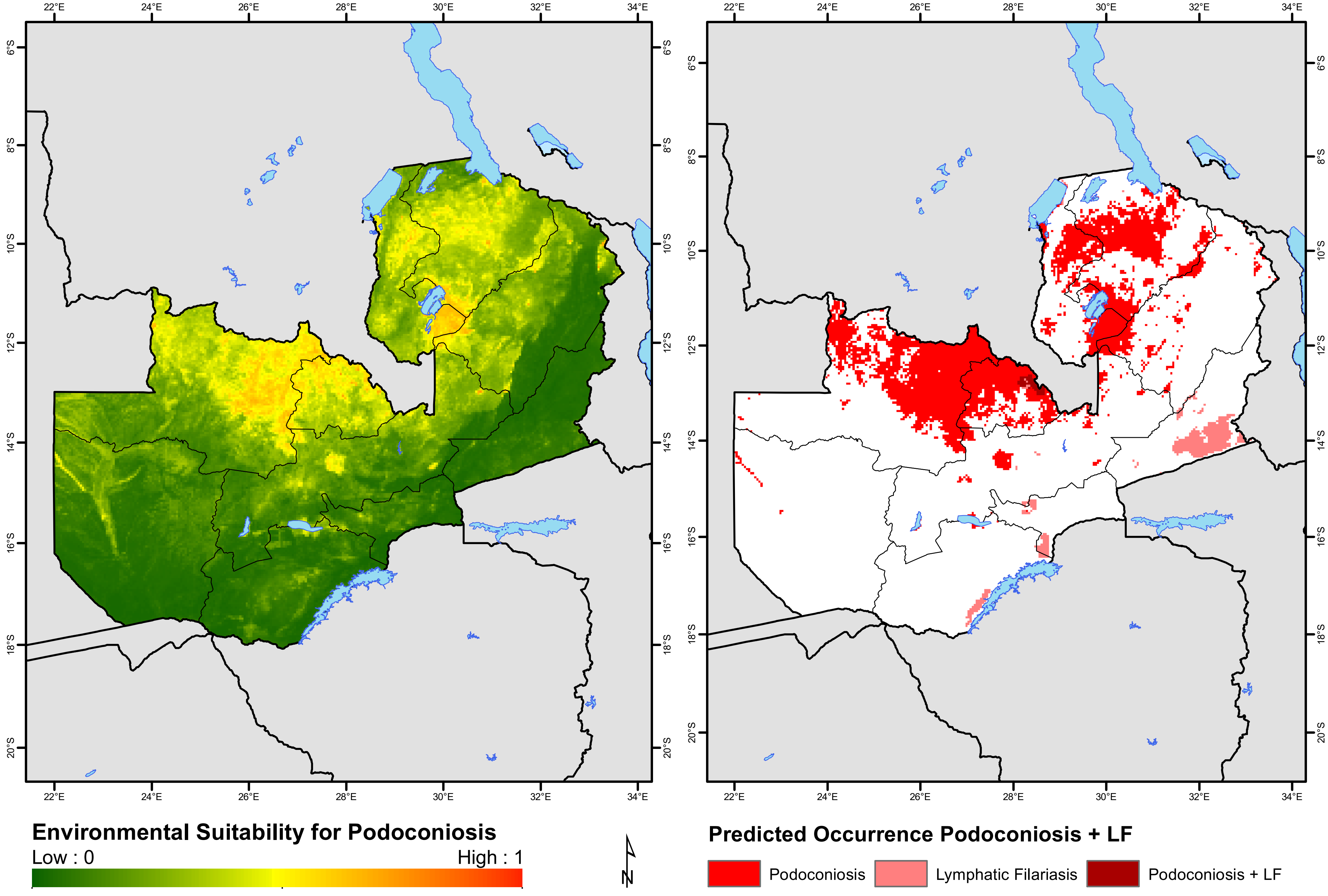
22°E
24°E
26°E
28°E
30°E
32°E
34°E
6°S
8°S
10°S
12°S
14°S
16°S
18°S
20°S
Environmental Suitability for Podoconiosis
Low : 0
High : 1
N
Predicted Occurrence Podoconiosis + LF
Podoconiosis
Lymphatic Filariasis
Podoconiosis + LF

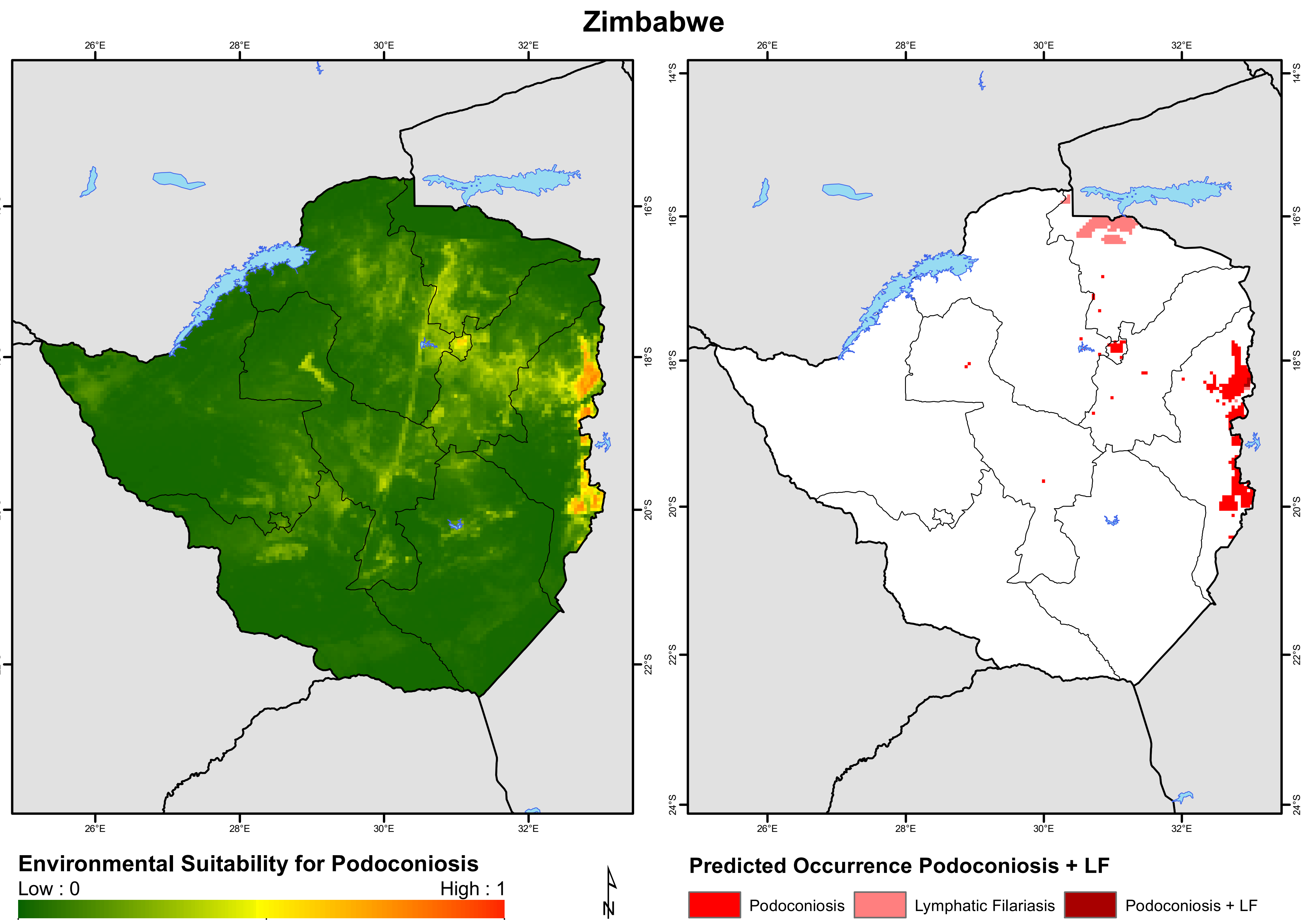
Zimbabwe
26°E
28°E
30°E
32°E
14°S
16°S
18°S
20°S
22°S
24°S
Environmental Suitability for Podoconiosis
Low : 0
High : 1
N
Predicted Occurrence Podoconiosis + LF
Podoconiosis
Lymphatic Filariasis
Podoconiosis + LF
